# *Pseudomonas syringae* pv. *actinidiae* (PSA) Isolates from Recent Bacterial Canker of Kiwifruit Outbreaks Belong to the Same Genetic Lineage

**DOI:** 10.1371/journal.pone.0036518

**Published:** 2012-05-09

**Authors:** Angelo Mazzaglia, David J. Studholme, Maria C. Taratufolo, Rongman Cai, Nalvo F. Almeida, Tokia Goodman, David S. Guttman, Boris A. Vinatzer, Giorgio M. Balestra

**Affiliations:** 1 Department of Science and Technologies for Agriculture, Forestry, Nature and Energy (DAFNE), University of Tuscia, Viterbo, Italy; 2 Department of Biosciences, University of Exeter, Exeter, Devon, United Kingdom; 3 Department of Plant Pathology, Physiology, and Weed Science, Virginia Tech, Blacksburg, Virgina, United States of America; 4 School of Computing, Federal University of Mato Grosso do Sul, Campo Grande, Brazil; 5 Center for the Analysis of Genome Evolution and Function, University of Toronto, Toronto, Ontario, Canada; The University of Hong Kong, China

## Abstract

Intercontinental spread of emerging plant diseases is one of the most serious threats to world agriculture. One emerging disease is bacterial canker of kiwi fruit (*Actinidia deliciosa* and *A. chinensis*) caused by *Pseudomonas syringae* pv. *actinidiae* (PSA). The disease first occurred in China and Japan in the 1980s and in Korea and Italy in the 1990s. A more severe form of the disease broke out in Italy in 2008 and in additional countries in 2010 and 2011 threatening the viability of the global kiwi fruit industry. To start investigating the source and routes of international transmission of PSA, genomes of strains from China (the country of origin of the genus *Actinidia*), Japan, Korea, Italy and Portugal have been sequenced. Strains from China, Italy, and Portugal have been found to belong to the same clonal lineage with only 6 single nucleotide polymorphisms (SNPs) in 3,453,192 bp and one genomic island distinguishing the Chinese strains from the European strains. Not more than two SNPs distinguish each of the Italian and Portuguese strains from each other. The Japanese and Korean strains belong to a separate genetic lineage as previously reported. Analysis of additional European isolates and of New Zealand isolates exploiting genome-derived markers showed that these strains belong to the same lineage as the Italian and Chinese strains. Interestingly, the analyzed New Zealand strains are identical to European strains at the tested SNP loci but test positive for the genomic island present in the sequenced Chinese strains and negative for the genomic island present in the European strains. Results are interpreted in regard to the possible direction of movement of the pathogen between countries and suggest a possible Chinese origin of the European and New Zealand outbreaks.

## Introduction

Infectious diseases of humans have emerged throughout history and spread over long geographic distances in short times. A striking example is the bubonic plague caused by *Yersinia pestis*, which rampaged through most of Europe in only six years after probably being imported into Europe from Caffa in Crimea in 1347. During the latest plague epidemic, *Y. pestis* spread from Hong Kong to all other continents besides Antarctica within 10 years. Whole genome sequencing and analysis of worldwide strains with genome-derived single nucleotide polymorphism (SNP) markers have now revealed the historic routes of transmission of *Y. pestis* [1]. But it is not only human pathogens whose geographic spread has impacted humans throughout history. For example, the intercontinental spread of the potato pathogen *Phytophtora infestans* from South America to North America, continental Europe and Ireland caused the Irish potato famine (1845–1852) [2].

The bacterial species *Pseudomonas syringae* comprises dozens of pathogens specialized on different crops. Although these pathogens are all members of the same species complex, they are differentiated in terms of host range. Many *P. syringae* pathogens are thought to cause disease only on single crop species. Based on their host range and the type of symptoms they cause, these pathogens are assigned to different intra-specific taxa, called pathovars [3]. In the case of *P. syringae* pv. *tomato*, the causal agent of bacterial speck disease of tomato, we recently used whole genome sequencing and SNP analysis to determine that a single clonal lineage of this pathogen has spread throughout the world starting in the 1970s [4]. A more recent example of an emerging *P. syringae* pathogen is a new genetic variant of *P. syringae* pv. *aesculi*, which was identified as the causal agent of a horse chestnut canker disease in the Netherlands and Belgium. The pathogen is currently making its way North through the UK decimating the horse chestnut population. Again, a single clonal lineage is responsible for this outbreak [5].

*Pseudomonas syringae* pv. *actinidiae* (PSA) is a pathovar that was first described in Japan in 1989 as the causal agent of bacterial canker of kiwifruit (*Actinidia deliciosa*) [6]. The disease consists of brown leaf spots with chlorotic haloes, brown discoloration of buds, cankers with exudates on trunks and twigs, and collapsed fruits [7]. The same disease was described in Hunan province in China as early as 1984/1985 [8] and soon spread to the provinces Sichuan, Anhui, and Shaanxi. Also in China, PSA was identified as causal agent [9], [10]. The disease was later found in Korea [11]. After an ephemeral outbreak in Italy in 1992 [12], a severe outbreak of bacterial canker started in Italy in 2008 [13]. During this latter outbreak the disease was initially associated with yellow flesh *A. chinensis* of which vegetative material had been imported from New Zealand and from China [14]. However, the disease was soon observed also on green flesh *A. deliciosa* cultivars [7]. By 2010 the disease had spread to Portugal and France on *A. deliciosa* as well as on *A. chinensis* [15], [16]. In Fall 2010, bacterial canker disease was also detected on *A. deliciosa* and *A. chinensis* in New Zealand [17], which together with Italy is the largest producer of kiwifruit worldwide (approximately 800,000 tons with a total commercial value of approximately € 2 billion). The New Zealand outbreak started in the Te Puke region and from there spread to the Tauranga region, and to areas of Katikati, Waihi and Whakatane during 2011 (www.kvh.org.nz). In 2011, PSA was also found in Spain [18], in Switzerland [19], in Chile [20], and in Australia [21]. Therefore, PSA is now threatening the viability of the global kiwifruit industry.

To prevent the further international spread of bacterial canker of kiwifruit it is imperative to determine the routes and modes of long distance transmission of PSA. *P. syringae* pathogens in general are known to be transmitted long distance by seed [22], [23] and vegetative material [24], [25]. Since aphids have been shown to transmit *P. syringae* in laboratory conditions, insect vectoring might also be a possibility [26]. Pollen, equipment, and people have specifically been suggested for PSA transmission [27], [28]. Recent research also revealed that the life cycle of *P. syringae* pathogens is linked with the water cycle since *P. syringae* bacteria have been isolated from rain, snow, snow pack, surface water, and irrigation water [29], [30]. Therefore, long distance movement of PSA through the atmosphere could be a possible mode of transmission.

Previous molecular work using multilocus sequence typing (MLST) and various molecular fingerprinting revealed that PSA isolates from Japan and Korea present a distinct genetic lineage, while strains from Italy and New Zealand represent a second genetic lineage [16], [31], [32], [33]. The highly virulent PSA isolates that cause canker in New Zealand are called PsaV, while a more distantly related PSA lineage, called PsaLV, is also present in New Zealand but is of low virulence and only causes leaf spotting [33].

No differences have so far been detected between Italian PSA strains and New Zealand PsaV strains. Consequently, these strains may belong to the same genetic lineage. The origin of these outbreaks remain unclear. The data available to date are unable to distinguish whether there was an Italian origin of the New Zealand outbreak, a common origin of both outbreaks in a third country, or a New Zealand origin of the Italian outbreak. Because kiwifruit originated in China (in the provinces of Hupeh, Szechuan, Kiangsi and Fukien in the Yangtze Valley of Northern China–latitude 31°N– and Zhejiang Province on the coast of eastern China [34]) and bacterial canker was already found in China in the 1980s, we hypothesized that PSA may have originated and co-evolved with the *Actinidia* genus in China. Moreover, since the bacterial canker outbreak in Italy in 2008 on *A. chinensis* followed the introduction of *A. chinensis* plant material from China and from New Zealand (where it was derived originally from Chinese plant material [14], [35]), a Chinese origin of the European and New Zealand outbreaks appears to be a possible hypothesis. To find first indications in regard to the geographic origin and possible routes and modes of transmission of PSA we sequenced and compared the genomes of two Japanese strains, one Korean strain, two Chinese strains, three Italian strains, and one Portuguese strain, and analyzed additional strains from Europe and New Zealand with markers derived from the whole-genome sequencing data.

## Results

### Strains and genomes

A draft genome sequence of the PSA pathotype strain MAFF 302091 (also referred to as strain M302091), isolated from *A. deliciosa* in Japan in 1984, and draft genome sequences of two Italian PSA strains, one isolated in 1992 and one isolated during the current epidemic, were recently published and compared to genomes of other *P. syringae* strains [36], [37]. To investigate the diversity that exists within PSA and to start reconstructing the phylogenetic relationship between PSA strains from different bacterial canker outbreaks that occurred in different years in different countries, we chose to generate draft genome sequences of two additional strains isolated in Japan, one strain isolated in Korea, two strains isolated in China (Province of Shaanxi), three strains isolated in Italy, and one strain isolated in Portugal. The genome of the pathotype strain of pathovar *theae* was also sequenced since it is the closest known relative of PSA [38]. Strain details are given in Table 1 and genome coverage and genome assembly data for each genome are summarized in Table 2, which also lists accession numbers of representative genomes submitted to Genbank. In short, genome coverage values ranged from 60 to 267 and assembled draft genomes consist of 431 to 513 contigs with N50 values ranging from 24,213 nt to 37,924 nt. These genome sequences were thus judged to be of sufficient quality to build phylogenetic trees based on alignments of the proteins these genomes encode.

**Table 1:** PSA Strains used in this study for genome sequencing or for analysis with genome-derived SNP markers.

strain name	species of isolation	cultivar	Country of isolation	site of isolation	year of isolation	source	type of analysis
Kw 41	*A. chinensis*	Hayward	Japan	Shizuoka	1984	Takikawa	genome
PA 459	*A. chinensis*	-	Japan	-	1988	CFBP	genome
Psa KN.2	*A. deliciosa*	-	Korea	-	1997	Koh	genome
CH2010–5	*A. chinensis*	Hongyang	China	Shaanxi	2010	DAFNE Unitus^b^	genome
CH2010–6	*A. chinensis*	Hongyang	China	Shaanxi	2010	DAFNE Unitus	genome
CH2010–7	*A. chinensis*	Hongyang	China	Shaanxi	2010	DAFNE Unitus	SNP
CFBP 7285	*A. chinensis*	Jin Tao	Italy	Veneto	2008	DAFNE Unitus	genome
CFBP 7286	*A. chinensis*	Hort16A	Italy	Lazio	2008	DAFNE Unitus	genome
CFBP 7287	*A. deliciosa*	Hayward	Italy	Lazio	2008	DAFNE Unitus	genome
346	*A. deliciosa*	Summer	Portugal	Entre Douro	2010	DAFNE Unitus	genome
2598^a^	*T. sinensis*	-	Japan	-	1974	NCPPB	genome
820	*A. deliciosa*	Erica	Portugal	Valença	2011	DAFNE Unitus	SNP
832	*A. deliciosa*	Hayward	Portugal	Santa Maria da Feira	2011	DAFNE Unitus	SNP
835	*A. deliciosa*	Hayward	Portugal	Vila Boa de Quires	2011	DAFNE Unitus	SNP
840	*A. deliciosa*	Hayward	Portugal	Quindadas Bocas Felgueiras	2011	DAFNE Unitus	SNP
846	*A. deliciosa*	Hayward (♂)	Portugal	Lago–Braga	2011	DAFNE Unitus	SNP
829	*A. chinensis*	JinTao	Spain	Pontevedra, Galicia	2011	DAFNE Unitus	SNP
830	*A. chinensis*	JinTao	Spain	Pontevedra, Galicia	2011	DAFNE Unitus	SNP
ISPaVe 019	*A. deliciosa*	Hayward	Italy	Lazio	1992	Loreti	SNP
1TO	*A. deliciosa*	Hayward	Italy	Piemonte	2010	DAFNE Unitus	SNP
16LT	*A. deliciosa*	Hayward	Italy	Lazio	2009	DAFNE Unitus	SNP
15ER	*A. deliciosa*	Hayward	Italy	Emilia Romagna	2011	Calzolari	SNP
490	*A. chinensis*	JinTao	Italy	Calabria	2010	DAFNE Unitus	SNP
770	*A. chinensis*	JinTao	Italy	Veneto	2011	DAFNE Unitus	SNP
1F	*A. chinensis*	JinTao	France	Aquitaine	2010	Anses^c^	SNP
3F	*A. deliciosa*	Hayward	France	Rhone Alpes	2010	Anses	SNP
5F	*A. deliciosa*	Hayward	France	Rhone Alpes	2010	Anses	SNP
14F	*A. chinensis*	Hort16A	France	Aquitaine	2010	Anses	SNP
16F	*A. deliciosa*	Summer	France	Aquitaine	2011	Anses	SNP
18839 (V)	*A. deliciosa*	Hayward	New Zealand	Bay of Plenty	2011	MAF^d^ New Zealand	SNP
18875 (V)	*A. deliciosa*	Hayward	New Zealand	Bay of Plenty	2011	MAF New Zealand	SNP

a pv. theae.

b Department for Agriculture, Forestry, Nature and Energy, University of Tuscia, Viterbo, Italy.

c French Agency for Food, Environment and Occupational Health Safety.

d Ministry of Agriculture and Forestry.

**Table 2:** Genome sequencing and assembly results for the nine sequenced PSA strains and the *P. syringae* pv. *theae* pathotype strain NCPPB 2598.

Strain Accession #^1^	Number of scaffolds	N50 (nt)	Longest scaffold (nt)	Total Length (nt)	Depth of genome coverage (X)
KW41 AGNP00000000	429	38609	136,008	5,926,530	194.3
PA459 AGNQ00000000	393	49,861	303,211	6,464,954	209.9
PsaKN.2	506	24437	113831	5921359	59.8
CH2010-5	497	27504	97099	6125814	141.5
CH2010-6 AGUH00000000	342	51971	198,200	6,199,322	231.6
CFBP 7285	508	26910	96897	6121804	80.1
CFBP 7286 AGNO00000000	352	43,501	139,438	6,142,224	82.2
CFBP 7287	510	25586	136839	6132952	119.2
346	501	27432	101295	6121411	80.4
NCPPB 2598^1^ AGNN00000000	218	7,937	242733	6,666,431	267.1

1 The versions described in this paper are the first versions xxxx01000000.

2 pathovar *theae*.

### Phylogenetic placement of PSA in respect to other sequenced P. syringae genomes

**Figure 1:**
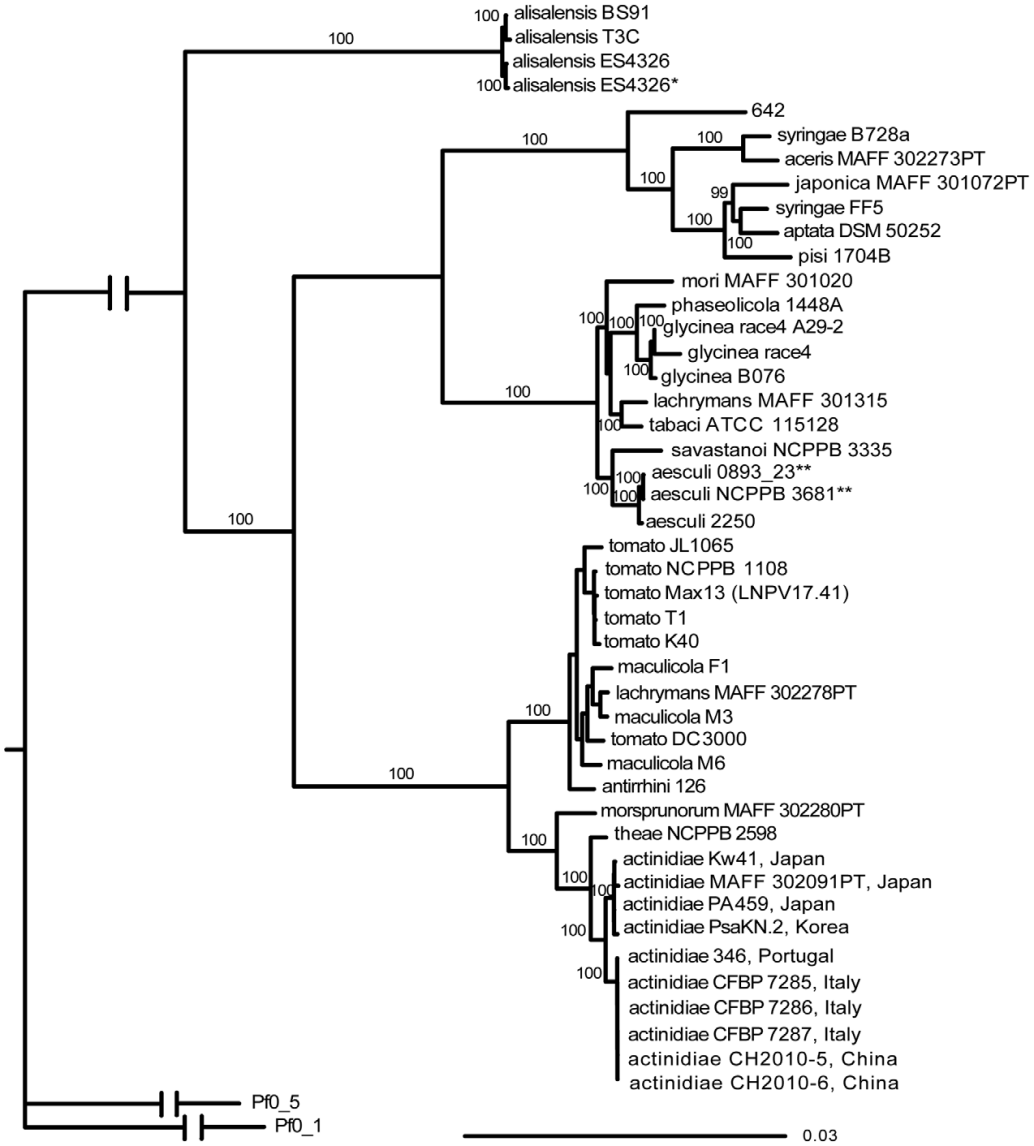
Phylogenetic tree placing *P. syringae* pv. *actinidiae* (PSA) within the *P. syringae* species complex. 1,186 proteins that are present exactly one time in each of the nine PSA strains and the *P. syringae* pv. *theae* pathotype strain NCPPB 2598 sequenced here and in each of 35 additional *P. syringae* strains for which genome sequences are available were aligned and concatenated. A maximum likelihood tree was then built using the two sequenced *P. fluorescens* strains Pf-0 and Pf5-1 as outgroups. Strains are labeled with pathovar names and strain names (genome accession numbers are listed in Table S1). Bootstrap values higher than 95 are shown at nodes. * Strain ES4326 is present twice since two stocks of this strain were sequenced separately. ** Strains 0893-23 and NCPPB 3681 are the same strain sequenced twice in two separate genome sequencing projects.

The newly sequenced PSA genomes and the genome of the pv. *theae* pathotype strain NCPPB 2598 were automatically annotated using the RAST server [39]. The predicted protein repertoires were then compared using OrthoMCL [40] with the protein repertoires of all other *P. syringae* strains for which genomes are either available from the National Center of Biotechnology Information (NCBI) or that were recently sequenced by us and collaborators (Table S1). Predicted protein repertoires of strains *P. fluorescens* Pf0-1 [41] and Pf5 [42] were also included. We then used the OrthoMCL results to identify those protein families that have exactly one member per genome in all *P. syringae* genomes. These 1,186 protein families were aligned and concatenated and used to build the phylogenetic tree shown in Figure 1.

The phylogenetic tree shows that PSA strains are separated into two main branches with one containing Korean and Japanese strains and the other containing the strains from the recent European outbreak and the Chinese strains. The existence of two PSA lineages is in agreement with previous results from multilocus sequence analysis (MLSA) of strains from Italy, New Zealand (PsaV strains), Japan, and Korea [31], [33]. However, since Chinese PSA strains were not previously subjected to molecular characterization this represents the first indication that European strains from the recent outbreak and Chinese strains belong to the same genetic lineage. The pathotype strain of pathovar *theae* was confirmed to be closely related to PSA followed by the pv. *morsprunorum* pathotype strain as next closest relative. The neighboring clade contains strains assigned to pathovars *tomato*, *maculicola*, *antirrhini*, and *lachrymans*. These two clades correspond to group 1 identified by MLSA [43] and genomospecies 3 identified by DNA-DNA hybridization [44] confirming recent results obtained by MLSA [38]. Other *P. syringae* pathovars clustered as previously determined by MLSA [38], [43], [45] and by a tree based on 324 proteins [36].

### European and Chinese strains are similar but not identical to each other

Since the phylogenetic tree in Figure 1 is based on alignment of proteins present in all considered *P. syringae* genomes and in the two *P. fluorescens* genomes, mutations in genes that are only present in PSA and the pathovar *theae* pathovar strain could not contribute to tree construction. Also, all synonymous mutations and mutations in non-coding regions were not considered since these do not affect protein sequences. Therefore, to obtain a higher phylogenetic resolution of the sequenced PSA strains we aligned reads of all newly obtained PSA strains against the assembled pv. *theae* genome and identified single nucleotide polymorphisms (SNPs) with respect to the pv. *theae* genome. Since SNP identification did not rely on the de novo assembly of the PSA genomes described above it was free of any potential assembly artifacts.

**Figure 2:**
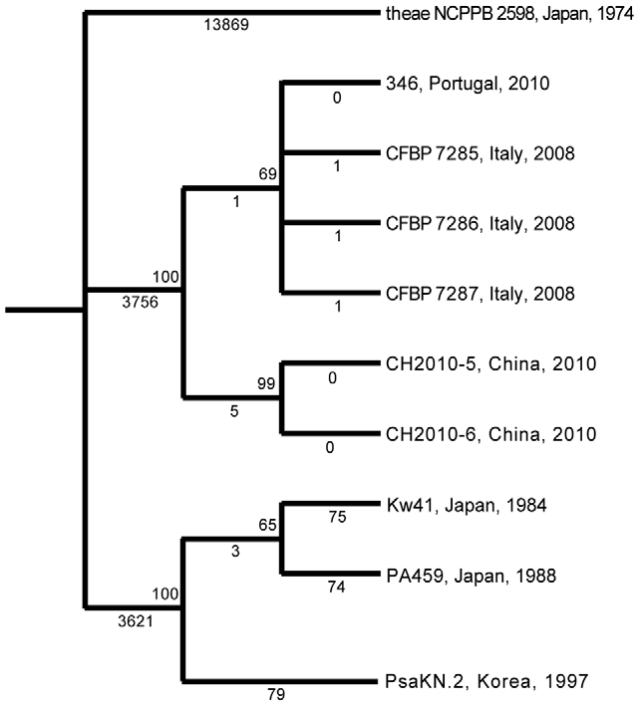
Neighbor Joining cladogram based on Single Nucleotide Polymorphisms (SNPs) identified between *P. syringae* pv. *actinidiae* (PSA) genomes and *P. syringae* pv. *theae*. Sequencing reads of nine PSA genomes were aligned against a draft genome of *P. syringae* pv. *theae* pathotype strain NCPPB 2598. A neighbor joining tree was built based on 21,494 SNPs so identified. Country and year of isolation are indicated for each strain. Bootstrap values based on 1000 bootstrap replicates are shown above nodes and number of SNPs compared to *P. syringae* pv. *theae* are shown underneath branches. Branches with less than 50% bootstrap support were collapsed. In the Japanese/Korean clade three SNPs group PsaKN.2 with PA459 and thus conflict with the branching pattern obtained in the tree. No SNPs conflict with the branching pattern obtained for the Chinese/European clade. A Bayesian tree was also constructed and had the same topology as the neighbor-joining tree.

To obtain highly reliable SNPs, we only used those regions of the pv. *theae* genome with a coverage depth of five or more for each sequenced PSA genome. Just over half the length (3,453,192 nt) of the pv. *theae* genome fulfilled this criterion. Over this length, a total of 21,494 SNPs (listed in Table S2) were identified that were supported by at least 95% of the reads at each nucleotide. Of these, 13,869 were invariant among all PSA genomes (i.e., the mutations leading to these SNPs occurred prior to the divergence of the PSA strains) and were not of interest. The remaining 7,625 SNPs were polymorphic among the PSA isolates (summarized in Table 3). A phylogenetic tree based on these SNPs is shown in Figure 2. Consistent with the whole genome tree, most SNPs distinguish the Korean/Japanese isolates from the European/Chinese isolates. Also the Japanese/Korean isolates represent a relatively diverse group with approximately 150 SNPs distinguishing isolates from each other. However, only 6 SNPs distinguish the two Chinese isolates (which were found to be identical to each other) from the European isolates. Only two SNPs distinguish the Italian isolates from each other and one SNP distinguishes each of them from the Portuguese strain.

**Table 3:** Number of SNPs distinguishing pairs of strains identified in 3,453,192 bp.

	*theae*	KW41	K2	PA459	CH2010–5	CH2010–6	346	7285	7286	7287
*theae*	0	-	-	-	-	-	-	-	-	-
KW41	17,571	0	-	-	-	-	-	-	-	-
K2	17,574	161	0	-	-	-	-	-	-	-
PA459	17,571	154	159	0	-	-	-	-	-	-
CH2010–5	17,633	7460	7465	7464	0	-	-	-	-	-
CH2010–6	17,633	7460	7465	7464	0	0	-	-	-	-
346	17,629	7456	7461	7460	6	6	0	-	-	-
CFBP 7285	17,630	7457	7462	7461	7	7	6	0	-	-
CFBP 7286	17,630	7457	7462	7461	7	7	6	1	0	-
CFBP 7287	17,630	7457	7462	7461	7	7	6	1	1	0

**Figure 3:**
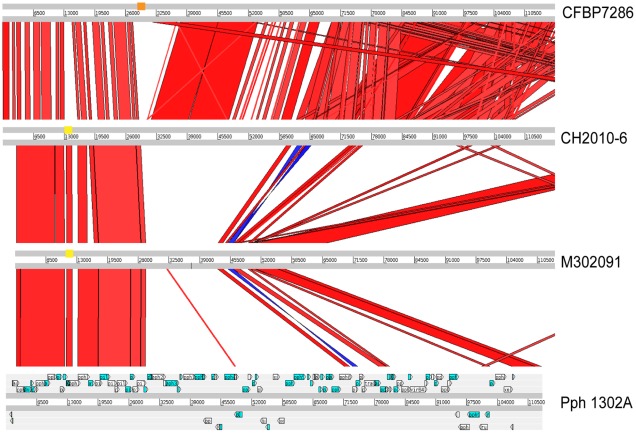
Alignment of the genomic island PPHGI-1 from *P. syringae* pv *phaseolicola* 1302A **(Accession # AJ870974) with similar islands in PSA strains CFBP7286 (Italy), CH2010-6 (China), and MAFF302091 (Japan).** Genomic regions of PSA strains were aligned with PPHGI-1 using BLASTN and visualized using the Artemis comparison tool [56]. Positions of PCR products that distinguish the island present in strain CH2010-6 and MAFF302091 from the island present in strain CFBP7286 are indicated by two yellow and one orange rectangle respectively. content differences within the islands of strains CFBP7286 and CH2010-6 compared to the MAFF302091 genome are listed in Table S5.

Considering the extremely high degree of DNA sequence identity between European and Chinese isolates, it is not surprising that these genomes are also almost identical in gene content. Tables S3 and S4 present a comprehensive list of genes that are differentially represented among the PSA strains or between PSA and pv. *theae*. The one exception is a genomic island similar to PPHGI-1, which was first described in *P. syringae* pv. *phaseolicola* [46]. Figure 3 shows an alignment of PPHGI-1 with similar islands in the PSA pathotype strain MAFF 302091 and in one representative each for the Chinese and European isolates. The Chinese and European isolates differ from each other in several regions within this island; however, these differences only affect putative mobile and hypothetical genes but not any known or predicted virulence genes (see Table S5).

**Table 4:** Position of all identified SNPs in the *P. syringae* pv. *theae* NCPPB 2598 genome (and primer sequences to amplify them) that distinguish European strains from each other and European strains from Chinese strains. Primer sequences to distinguish between the European and Asian version of the PPHGI-1 island are also listed. Results obtained by PCR and Sanger sequencing are indicated.

scaffold, contig, position	Primer sequences F and R	Europe/NZ	China	Japan/Korea/Italy1992
592, 29, 1444	GTGGCGGTTATCTGTACGC	C (T only in 7285)	C	C
	CTTTTCCTTGACCAGCGTGT			
876, 5, 198	TGGCCATGATCAAGTGTCTG	C (A only in 7286)	C	C
	AAGAAGTCAAAGCCCTGCTC			
163, 3, 12532	GGAGAAAGCTTCGTGGTCAG	C (T only in 7287)	C	C
	ACTCTGCAGCCATTCCAGAT			
176, 5, 6360	GGTCACCAGTACAACGCTCA	G	A	G
	ACCAGCCAATCCTTTACGTG			
237, 121, 1890	CTTGTCGTTCCATTCCATCC	C	A	C
	GGTATCGACAACGCCTCTTA			
398, 19, 3855	ACGAAGGCCTGTACCGAAGT	T	A	T
	CGACGGTCAGGAAGGTTATC			
452, 21, 2795	CCTGCGCTGACTGAAATCAT	G	A	G
	GACGTCATGACCTTGAGTTGTT			
911, 61, 832	GATAACCGCCCACCTGATAG	TGA	AGA	TTG
	ACGGCTATTACCCGCTCAAC			
190, 7, 4380	GTGACCGACTCGCTGAAAAG	C	T	T
	CGGATGTTCTACATGCGCTAC			
PPHGI-1 Europe F	TGGTGATCGTCTGGATGTGT	+/−	−	−
PPHGI-1 Europe R	ATTATGCTCCTGGCTCATGG			
PPHGI-1 Asia F	ACTGAATTGAATGAGGCGGTTA	−/+	+	+
PPHGI-1 Asia R	GTCATGAATCAATGAGCTCAAAGTG			

### Comparison of PSA strains with genome-derived markers reveals minimal but informative differences between isolates from Europe, New Zealand, and China

Since our ultimate goal is to determine the origin of the PSA lineages causing the current bacterial canker outbreaks in Europe and New Zealand, we designed genome-derived markers to analyze additional isolates beyond the strains for which we obtained draft genome sequences. In particular, we designed PCR primers up- and downstream of the six SNPs that distinguish the European strains from the Chinese strains and up- and downstream of the SNPs that distinguished individual Italian isolates from each other and from the Portuguese and Chinese isolates. One primer pair encompassed also two SNPs that distinguish the European/Chinese lineage from the Korean/Japanese lineage making this primer pair particularly useful in typing unknown PSA isolates. The typing of these eleven SNPs showed that all recent isolates from Italy, France, Spain, Portugal, and New Zealand (PsaV) were identical to each other and to the sequenced European isolates. None of these isolates shared the SNPs unique to either one of the sequenced Italian isolates. The PSA strain isolated in Italy in 1992 [12], [37] was the only strain that was indistinguishable in all analyzed SNP loci from the Korean/Japanese isolates confirming that this earlier Italian outbreak is not related to the recent bacterial canker outbreak in the same country.

We also designed two pairs of primers for distinguishing between the different PPHGI-1-like islands. One primer pair was designed on a small region of the PPHGI-1 island present only in the sequenced European strains but not in the sequenced Chinese and Japanese/Korean strains (the “European amplicon”), while a second primer pair was designed on a small region of the PPHGI-1 island present only in the Chinese and Japanese/Korean strains but not in the sequenced European strains (the “Asian amplicon”; see Figure 3 for location of the targeted regions). Interestingly, while all European isolates amplified the European amplicon, the two New Zealand PsaV isolates amplified only the Asian amplicon although they were identical to the European isolates–and not the Chinese isolates–at the analyzed SNP loci. Sequencing of the amplicons obtained from the New Zealand PsaV isolates showed 100% identity to the corresponding region of the two sequenced Chinese isolates (which are different in this region from the Japanese/Korean isolates).

## Discussion

Until the advent of next generation genome sequencing, it was very often challenging to track the descent and dissemination of genetically monomorphic bacterial pathogens. Whole genome re-sequencing and high resolution SNP mapping was required to precisely reconstruct the spread of pathogens such as *B. anthracis* [47] and *Y. pestis* [1] around the globe. Since MLSA could not distinguish PSA isolates from the recent kiwifruit bacterial canker outbreaks in Italy and New Zealand [33] these outbreaks also appeared to be caused by a genetically monomorphic pathogen. Therefore, we decided to use Illumina (Solexa) sequencing [48], to obtain further insight into the origin of the current kiwifruit bacterial canker epidemics.

We show that when multiplex sequencing eight *P. syringae* genomes per flowcell channel on an Illumina Genome Analyzer GAIIx it is possible to obtain draft genomes of ∼60x read depth that are of sufficient quality for SNP analysis. We were able to identify 1,186 core protein orthologs present exactly one time in each of the 10 sequenced genomes and in 35 other *P. syringae* strains with draft or finished genomes. A strongly supported core genome phylogenetic tree based on the alignment of these protein sequences was then built (most branches have bootstrap values greater than 99), which allowed us to place the sequenced PSA isolates into a precise phylogenetic context within the *P. syringae* species complex. This approach distinguished the four PSA isolates from Japan and Korea from the PSA isolates from Europe and China. However, it was not sufficient to distinguish PSA isolates from Europe and China from each other.

The most reliable approach to identifying SNPs among closely related bacterial strains is to perform a reference-based assembly of individual sequencing reads against a well-characterized genome [49]. This approach permitted the use of stringent read quality, sequencing depth, and data congruence cutoffs. In our case we implemented a minimum depth cutoff of five-fold coverage and a data congruence standard that all SNPs must be supported by 95% of all reads. We validated these stringency criteria by confirming the presence of eleven SNPs via PCR amplification of the appropriate regions and Sanger sequencing.

The core genome phylogenetic analysis shown in Figure 1 and the SNP-based tree shown in Figure 2 confirm previous reports that the Japanese and Korean PSA bacteria are clearly distinct from those that cause bacterial canker in Europe since 2008 [16], [31], [32]. Moreover, the latter are almost identical to the two sequenced Chinese isolates. Comparison of the recently published genome sequences of two Italian isolates, one from the 1992 outbreak and one from the current outbreak [37], with our genome sequences confirm that the Italian 1992 outbreak was caused by members of the Japanese/Korean lineage. The CRA-FRU 8.43 isolate from the current Italian outbreak sequenced by Marcelletti and co-workers [37] is instead identical to the Italian isolates CFBP7285, 7286 and 7287 sequenced here (data not shown). Approximately 7000 SNPs distinguish the Japanese/Korean PSA lineage from the European/Chinese lineage. Moreover, PSA strains as a whole cluster in a monophyletic clade with pv. *theae* as the most closely related outgroup strain. These data suggest that the two lineages diverged from a common ancestor rather than one being derived from the other. The higher diversity of the pathogen population in Japan and Korea compared to the pathogen population in Europe suggests a more recent origin of the European lineage, but of course this assumes that there has been no sampling bias. The number of SNPs among Japanese and Korean isolates (∼150 in 3,426,298 bp) is in the order of magnitude found between pairs of pv. *tomato* strains (∼53 to 183 in 3,543,009 bp) for which we recently estimated a divergence time from their most recent common ancestor between 283 and 1415 years [4]. Interestingly, a similar number of SNPs (97 in 4,367,867 bp) was identified between a genome of a medieval *Y. pestis* strain estimated to have lived in approximately the year 1350 and the genome of a current-day *Y. pestis* strain [50] further supporting a divergence time of Japanese and Korean strains from their most recent common ancestor in the order of hundreds of years.

While previous molecular analyses of PSA did not include strains from China, the country of origin of the genus *Actinidia* and the country where bacterial canker of kiwifruit was first described [8], here we sequenced two isolates from the Chinese province of Shaanxi, which proved to have identical genome sequences. Only 6 SNPs distinguish the Chinese isolates from the European isolates, while the European isolates differ from each other by one or two SNPs. These data indicate a very recent common ancestor for the Chinese-European lineage. For comparison, three *P. syringae* pv *aesculi* strains from the bleeding horse chestnut canker outbreak in Europe that was first noticed in 2002/2003 were isolated in 2006 and 2008 in the UK and differ by 3 SNPs in approximately 3 million bp. Based on these data, the three *P. syringae* pv. *aesculi* strains appear to have accumulated 3 SNPs in approximately 10 years. Therefore, assuming similar population dynamics and a similar mutation rate for PSA, the sequenced European and Chinese isolates may have diverged from their most recent common ancestor in not more than a few dozen years. However, only sequencing additional PSA isolates collected in different years will make it possible to apply Bayesian statistics to estimate divergence times for PSA as was recently done for *Helicobacter pylori* [51] and a clonal lineage of *Staphylococcus aureus* [52].

Further sampling and genomic analysis of PSA in all countries affected by bacterial canker of kiwi fruit will be necessary to conclusively determine the origin of this outbreak and to reconstruct with high resolution the path of dissemination of PSA. However, if we assume that the isolates analyzed so far are representative of the current PSA populations in the different countries, we propose the following preliminary hypothesis. PSA was transferred from China to Europe possibly via contamination of imported vegetative material of *A. chinensis* cultivars. Italy was most likely the point of entry of PSA into Europe given its large kiwifruit industry and since this is where the strain was first identified. These conclusions are supported by the longer branch leading to the two Chinese isolates (See Figure 2) and the relative homogeneity of the European strains despite their isolation from different geographic locations. This lack of polymorphism among European isolates is most likely due to a founder event (establishment of a new population by a small subsample of the source population; in the case of PSA, possibly a small number of bacteria present in a shipment of vegetative material from China to Italy). A founder event necessarily results in a dramatic reduction in the effective population size of the new population characterized by a loss of genetic variation. This scenario would be strongly supported by the identification of greater genetic polymorphism in the source Chinese population, and while we do find that the Chinese lineage is more divergent, we do not see the expected level of polymorphism. This discrepancy can be explained by either sampling bias or by a selective sweep that purged genetic variation among the Chinese PSA population. The former explanation has to be considered the null model, particularly since the two Chinese PSA isolates in this study were collected in the same year and location.

SNP analysis performed on PSA isolates from Italy, Portugal, Spain, France, and New Zealand (PsaV isolates) revealed that all isolates were identical to the sequenced European isolates at all 11 SNP loci (excluding the analyzed 1992 Italian isolate that was found to be identical to the Japanese/Korean lineage). While full genome sequencing of these strains would be preferable to a limited SNP analysis, the very recent timeframe of these outbreaks, in particular, the conclusion by the New Zealand Ministry of Agriculture and Forestry that the New Zealand outbreak is due to a single point of introduction not earlier than 2008 [28] makes it unlikely that much additional genetic variation has accumulated in the PSA populations in either Europe or New Zealand beyond that revealed by our original genome sequencing.

While our current genomic data suggest a possible Chinese origin of the European outbreak, we only have data obtained with genome-derived markers for the New Zealand outbreak. The origin of the New Zealand outbreak will thus remain an open question until complete genome sequences from New Zealand strains become available. Nevertheless, data to date show that New Zealand PsaV isolates are indistinguishable from Italian PSA isolates at 19 loci and are clearly distinct from isolates of the Japanese/Korean lineage [33]. We were able to show here that the European and New Zealand isolates carry the same putative ancestral SNP alleles for five of the six loci that distinguish European from Chinese isolates. If the PSA source population was indeed in China, then this supports a larger effective size of the Chinese PSA population compared to the European or New Zealand populations and, consequently, the previously discussed founder event.

The finding that the New Zealand PsaV isolates carry the version of the PPHGI-1 island present in the sequenced Chinese isolates but not the version present in the European isolates is potentially very important. These data indicate that New Zealand may have been colonized by PSA independently from Europe, or that there has been very recent replacement of part or all of this genomic island in New Zealand PsaV strains. It must be emphasized that this conclusion is preliminary and requires further investigation, yet may be the key to identifying the source of the recent New Zealand outbreak.

The commercial shipping of yellow flesh kiwifruit vegetative material between China, Europe, and New Zealand during the years preceding the bacterial canker outbreaks provides a possible transmission mechanism of PSA. This hypothesis is also supported by the observation that the first Italian outbreak occurred on yellow flesh cultivars. However, a recent investigation into possible modes of PSA transmission by the New Zealand Ministry of Agriculture and Forestry deemed this scenario unlikely since all vegetative material imported into New Zealand was kept in quarantine for several years and did not show any canker symptoms [28]. Moreover, yellow flesh cultivars are more susceptible to PSA than green flesh cultivars and this could also be the reason why the disease first appeared in yellow flesh cultivars. Therefore, other modes of transmission besides contaminated vegetative material cannot be excluded at this point, for example, dissemination via pollen, equipment, or people [27], [28]. Also long distance movement of PSA through the atmosphere and the water cycle or by insects remain valid hypotheses [26], [29], [30].

In conclusion, we have shown that genome sequencing and genome-derived markers are excellent tools to readily investigate the epidemiology of a genetically monomorphic plant pathogen like PSA. Future sampling of the diversity of the pathogen at its likely geographic origin and in all countries where the disease has now been described, followed by genome sequencing and in-depth genome comparisons can be expected to give the necessary insight into pathogen movement to contain as efficiently as possible the current bacterial canker epidemic on kiwi fruit. Lessons learned from PSA should also help to avoid future epidemics of emerging plant diseases caused by other bacterial pathogens.

## Materials and Methods

### Bacterial strains, growth, and DNA extraction

Bacterial strains are listed in Table 1. Bacteria were grown on NSA agar for 72 hrs at 26°C. DNA was isolated using the PureLink™ Genomic DNA Kit (Invitrogen, Carlsbad, CA, USA) following the manufacturer's instructions for Gram-negative bacteria.

### Genome sequencing

5 µg of each genomic DNA was sheared to approximately 300 bp size using the Covaris S2 (Covaris, Woburn, MA, USA) with the following conditions: Intensity 4; Duty cycle 10%; 200 bursts per cycle; and 120s total time. End repair and A-tailing were performed as described for standard Illumina DNA fragment libraries. Barcoded adaptors were independently ligated to each sample after which equal volumes of the samples were pooled and size selected on a 1% 0.5X TAE gel. A band around 400 bp size was excised. The purified band was subjected to 16 rounds of amplification following Illumina's protocol. A 400 bp band was size selected from the amplified product on a second 1% 0.5X TAE agarose gel to remove residual adaptors. The final product was quantitated using the Bioanalyzer 2100 DNA 1000 chip (Agilent, Santa Clara, CA. USA) and the Qubit Fluorometer using the dsDNABR kit (Life Technologies, Burlington, ON. Canada). PE sequencing was performed in one channel for 76 cycles on an Illumina GAIIx with PE module. The data was analyzed using the Illumina OLB pipeline v1.8. The total cluster number was 49,050,122 with 35,334,887 PF clusters or 72%.

### Construction of a P. syringae tree based on concatenated protein sequences

Sequencing reads were quality trimmed and assembled in CLC Genomics Workbench version 4 (Arhus, Denmark). “Trim using Quality Scores” was set to 0.05 and “Trim ambiguous nucleotide” was set to 2. The *de novo* assembly was performed using the trimmed reads. Assemblies were annotated using the RAST server (http://rast.nmpdr.org/). OrthoMCL [40] was run on the ten new genomes and 35 other available *P. syringae* genomes (Table S1) and the two *P. fluorescens* genomes Pf0_1 and Pf0_5. 1,186 ortholog families (out of a total of 13,937 ortholog families) were found with exactly one protein in each ingroup genome (all *P. syringae* genomes) and at most one in each outgroup genome (*P. fluorescens* genomes). These 1,186 families were aligned using Muscle [53] and then concatenated forming an alignment with 320,401 columns. Gblocks [54] was used to eliminate poorly aligned columns and divergent regions of the alignment. The maximum likelihood tree shown in Figure 1 was built using RAxML [55] with PROTGAMMAWAGF as model and 100 bootstrap trees.

### Genome-wide SNP detection

For each genome-wide Ilumina sequence dataset, we aligned the sequence reads against the reference genome sequence using BWA 0.5.5 (Li & Durbin, 2009). We then used SAMtools 0.1.16 (Li et al., 2009) to convert the alignments into Pileup format, which contains depths of coverage at each genomic position as well as the identities of the nucleotides aligned at each genomic position. We used the Pileup file to identify genomic positions at which depth of coverage was at least 5 and a consensus of at least 95% of the aligned reads at the position supported a SNP between the Illumina sequence reads and the reference sequence; all other positions were considered ambiguous and were not considered for SNP-calling. We constructed a multiple sequence alignment consisting of concatenations of genomic positions in which we had identified a SNP in at least one strain.

### Phylogenetic SNP tree construction

A Neighbor Joining (NJ) tree was built in PAUP version 4.0 (http://paup.csit.fsu.edu/) based on all SNPs that distinguish PSA strains from each other and using *P. syringae* pv. *theae* strain NCPPB2598 as outgroup. 1000 bootstrap replicates were performed.

### SNP analysis by PCR and Sanger sequencing

Primers were designed based on the genome of *P. syringae* pv. *theae* NCPPB 2598 using the program Primer3 (http://frodo.wi.mit.edu/primer3/) and are listed in Table 4. PCR reactions were done in 25 µL using a premixed ready-to-use solution containing a modified Taq DNA polymerase, dNTPs, MgCl_2_ and reaction buffers at optimal concentrations (GoTaq® Colorless Master Mix, Promega), upstream and downstream primers at a final 1.0 μM concentration each, and about 40 ng of DNA template. The thermic profile for amplification consisted in an initial denaturation step at 94°C for 5 min, followed by 30 cycles at 94°C for 30 s, 58°C for 30 sand 72°C for 30 s, and by a last elongation step for 7 min. PCR products were initially verified on an 1.5% agarose gel under UV light, purified, and then sequenced on both strands using the same primers used for amplification by the custom DNA sequencing service of Macrogen Europe (Amsterdam, The Netherlands). Potential ambiguities were resolved through close inspection of the corresponding chromatogram (Chromas Lite version 2.01) or by re-sequencing. Sequences were aligned using CLUSTALX software (version 2.0.11).

## Supporting Information

Table S1 *P. syringae* genomes previously sequenced and used for the construction of the phylogenetic tree shown in Figure 1.
(XLSX)

Table S2 Complete list of SNPs distinguishing the sequenced PSA genomes from each other and from *P. syringae* pv. *theae* NCPPB 2598.
(XLSX)

Table S3 Comparison of gene content among PSA isolates based on aligning reads against the reference genome of *Psa* NCPPB3739 (GenBank:AFTH01000000).
(PDF)

Table S4 Comparison of gene content among PSA isolates based on aligning reads against the reference genome of *Psa* NCPPB3871 (GenBank:AFTF01000000).
(PDF)

Table S5 Gene contents of isolates CFBP7286 (Europe) and CH2010-6 (China) in the region of the PPHGI-1- like island based on aligning reads against the reference genome of PSA MAFF302091.
(PDF)
