# Supplementary material for: Pseudomonas syringae pv. actinidiae (PSA) Isolates from Recent Bacterial Canker of Kiwifruit Outbreaks Belong to the Same Genetic Lineage
Source: PLoS One. 2012 May 9;7(5):e36518. doi: 10.1371/journal.pone.0036518 (PMC3348921; doi:10.1371/journal.pone.0036518)
Supplement: Table S3 — Comparison of gene content among PSA isolates based on aligning reads against the reference genome of Psa NCPPB3739 (GenBank:AFTH01000000). (PDF) [file pone.0036518.s003.pdf]

Supplementary Table 3. Comparison of gene content among *Psa* isolates based on aligning reads against the reference genome of *Psa* NCPPB3739 (GenBank:AFTH01000000).

Gene	Location	346	7285	7286	7287	K2	KW41	PA459	CH2010-5	CH2010-6	NCPPB2598
As predicted by RAST (http://http://rast.nmpdr.org/)	(GenBank accession: start-end)	Portugal	Italy	Italy	Italy	Korea	Japan	Japan	China	China	Japan (theae)
Transposase and inactivated derivatives;Note=Transposase and inactivated derivatives	AFTH01000611.1: 4152-5201	0.03	0.03	0.03	0.03	1.00	1.00	0.03	0.03	0.03	0.03
Name=Integrase Integrase;Note=Integrase	AFTH01000608.1: 7182-8417	0.05	0.05	0.04	0.05	1.00	1.00	1.00	0.11	0.11	0.00
FIG00960122: hypothetical protein;Note=FIG00960122: hypothetical protein	AFTH01000608.1: 8689-10047	0.93	0.94	0.94	0.95	1.00	1.00	1.00	0.96	0.96	0.00
putative integrase protein;Note=putative integrase protein	AFTH01000603.1: 481-2139	1.00	1.00	1.00	1.00	0.00	0.14	1.00	1.00	1.00	1.00
Name=DNA_invertase DNA-invertase;Note=DNA-invertase	AFTH01000603.1: 2123-2683	1.00	1.00	1.00	1.00	0.00	0.36	1.00	1.00	1.00	1.00
hypothetical protein;Note=hypothetical protein	AFTH01000588.1: 107-1519	0.01	0.01	0.02	0.01	1.00	1.00	1.00	0.12	0.12	0.02
Sucrose specific transcriptional regulator CscR2C LacI family;Note=Sucrose specific transcriptional regulator CscR2C LacI fa	AFTH01000580.1: 240-1187	1.00	1.00	1.00	1.00	0.00	1.00	1.00	1.00	1.00	1.00
Transducer protein car;Note=Transducer protein car	AFTH01000580.1: 1306-7014	1.00	1.00	1.00	1.00	0.00	1.00	1.00	1.00	1.00	1.00
Methyl-accepting chemotaxis protein;Note=Methyl-accepting chemotaxis protein	AFTH01000574.1: 3273-3647	0.82	0.96	1.00	0.97	0.00	0.00	1.00	1.00	1.00	1.00
hypothetical protein;Note=hypothetical protein	AFTH01000574.1: 3705-5111	0.98	0.98	1.00	1.00	0.00	0.00	1.00	1.00	1.00	1.00
Aconitase B;Note=Aconitase B	AFTH01000574.1: 5121-5450	1.00	1.00	1.00	1.00	0.00	0.16	1.00	1.00	1.00	1.00
Putative heat shock protein YegD;Note=Putative heat shock protein YegD	AFTH01000556.1: 2445-4010	1.00	1.00	1.00	1.00	1.00	1.00	1.00	1.00	1.00	0.00
hypothetical protein;Note=hypothetical protein	AFTH01000556.1: 4560-4724	1.00	1.00	1.00	1.00	1.00	1.00	0.90	1.00	1.00	0.00
plasmid stability protein StbC;Note=plasmid stability protein StbC	AFTH01000552.1: 2222-2344	1.00	1.00	1.00	1.00	1.00	1.00	1.00	1.00	1.00	0.00
plasmid stability protein StbB;Note=plasmid stability protein StbB	AFTH01000552.1: 2341-2487	1.00	1.00	1.00	1.00	1.00	1.00	1.00	1.00	1.00	0.00
FIG00959382: hypothetical protein;Note=FIG00959382: hypothetical protein	AFTH01000552.1: 4851-5771	1.00	1.00	1.00	1.00	1.00	1.00	1.00	1.00	1.00	0.00
SanC2C putative;Note=SanC2C putative	AFTH01000552.1: 8388-8759	1.00	1.00	1.00	1.00	1.00	1.00	1.00	1.00	1.00	0.00
hypothetical protein;Note=hypothetical protein	AFTH01000551.1: 588-713	1.00	1.00	1.00	1.00	1.00	1.00	1.00	1.00	1.00	0.00
FIG00963107: hypothetical protein;Note=FIG00963107: hypothetical protein	AFTH01000540.1: 11799-13172	1.00	1.00	1.00	1.00	1.00	1.00	1.00	1.00	1.00	0.00
hypothetical protein;Note=hypothetical protein	AFTH01000527.1: 1288-1500	1.00	1.00	1.00	1.00	0.00	0.00	1.00	1.00	1.00	0.00
ABC-type polar amino acid transport system2C ATPase component;Note=ABC-type polar amino acid transport system2C A	AFTH01000517.1: 34267-35064	1.00	1.00	1.00	1.00	1.00	1.00	1.00	1.00	1.00	0.01
ABC-type amino acid transport/signal transduction systems2C periplasmic component/domain;Note=ABC-type amino acid	AFTH01000517.1: 36446-36799	1.00	1.00	1.00	1.00	1.00	1.00	1.00	1.00	1.00	0.00
hypothetical protein;Note=hypothetical protein	AFTH01000764.1: 1553-2389	1.00	1.00	1.00	1.00	1.00	1.00	1.00	1.00	1.00	0.00
ABC-type amino acid transport/signal transduction systems2C periplasmic component/domain;Note=ABC-type amino acid	AFTH01000517.1: 37018-37317	1.00	1.00	1.00	1.00	1.00	1.00	1.00	1.00	1.00	0.00
membrane protein2C putative;Note=membrane protein2C putative	AFTH01000764.1: 2515-3042	1.00	1.00	1.00	1.00	1.00	1.00	1.00	1.00	1.00	0.00
site-specific recombinase2C phage integrase family;Note=site-specific recombinase2C phage integrase family	AFTH01000508.1: 1139-2149	1.00	1.00	1.00	1.00	1.00	1.00	1.00	1.00	1.00	0.00
Serine protease;Note=Serine protease	AFTH01000508.1: 2127-3182	0.08	0.07	0.08	0.08	1.00	1.00	1.00	0.08	0.08	0.00
TonB-dependent receptor%3B Outer membrane receptor for ferrienterochelin and colicins;Note=TonB-dependent recept	AFTH01000505.1: 53-2452	0.02	0.02	0.02	0.02	1.00	1.00	1.00	0.09	0.09	0.02
avirulence protein;Note=avirulence protein	AFTH01000500.1: 16469-16915	1.00	1.00	1.00	1.00	1.00	1.00	1.00	1.00	1.00	0.00
hypothetical protein;Note=hypothetical protein	AFTH01000499.1: 7020-7157	1.00	1.00	1.00	1.00	1.00	1.00	1.00	1.00	1.00	0.00
Conjugative transfer protein PilN in PFGI-1-like cluster;Note=Conjugative transfer protein PilN in PFGI-1-like cluster	AFTH01000498.1: 8991-10727	0.04	0.00	0.04	0.13	1.00	1.00	1.00	0.13	0.13	1.00
Conjugative transfer protein PilL in PFGI-1-like cluster;Note=Conjugative transfer protein PilL in PFGI-1-like cluster	AFTH01000498.1: 10724-11869	0.03	0.04	0.04	0.17	1.00	1.00	1.00	0.34	0.28	1.00
DNA/RNA helicase in PFGI-1-like cluster;Note=DNA/RNA helicase in PFGI-1-like cluster	AFTH01000498.1: 13413-15371	0.04	0.04	0.04	0.04	1.00	1.00	1.00	0.87	0.86	0.72
Conjugative transfer protein TrbJ;Note=Conjugative transfer protein TrbJ	AFTH01000760.1: 15126-15449	0.05	0.08	0.06	0.08	0.08	1.00	0.08	0.09	0.09	0.00
Protein involved in meta-pathway of phenol degradation;Note=Protein involved in meta-pathway of phenol degradation	AFTH01000488.1: 27352-28245	0.04	0.04	0.05	0.05	1.00	1.00	1.00	0.05	0.05	0.86
FIG045374: Type II restriction enzyme2C methylase subunit YeeA;Note=FIG045374: Type II restriction enzyme2C methylas	AFTH01000488.1: 28230-30302	1.00	1.00	1.00	1.00	1.00	1.00	1.00	1.00	1.00	0.00
major facilitator family transporter;Note=major facilitator family transporter	AFTH01000486.1: 15976-16947	1.00	1.00	1.00	1.00	1.00	1.00	1.00	1.00	1.00	0.00
3-oxoacyl-[acyl-carrier-protein] synthase2C KASIII;Ontology_term=KEGG_ENZYME:2.3.1.41;Note=3-oxoacyl-[acyl-carrier-p	AFTH01000486.1: 16990-17985	1.00	1.00	1.00	1.00	1.00	1.00	1.00	1.00	1.00	0.00
bacterial transferase2C hexapeptide repeat protein;Note=bacterial transferase2C hexapeptide repeat protein	AFTH01000486.1: 18012-18653	1.00	1.00	1.00	1.00	1.00	1.00	1.00	1.00	1.00	0.00
3-oxoacyl-[acyl-carrier protein] reductase;Ontology_term=KEGG_ENZYME:1.1.1.100;Note=3-oxoacyl-[acyl-carrier protein]	AFTH01000486.1: 18650-19411	1.00	1.00	1.00	1.00	1.00	1.00	1.00	1.00	1.00	0.00
FIG00955995: hypothetical protein;Note=FIG00955995: hypothetical protein	AFTH01000486.1: 19466-19672	1.00	1.00	1.00	1.00	1.00	1.00	1.00	1.00	1.00	0.00
hypothetical protein;Note=hypothetical protein	AFTH01000446.1: 470-601	1.00	1.00	1.00	1.00	1.00	1.00	1.00	1.00	1.00	0.00
transcriptional regulator2C LysR family;Note=transcriptional regulator2C LysR family	AFTH01000756.1: 251-1075	1.00	1.00	1.00	1.00	0.00	1.00	1.00	1.00	1.00	1.00
TRAP-type C4-dicarboxylate transport system2C periplasmic component;Note=TRAP-type C4-dicarboxylate transport syste	AFTH01000407.1: 1095-1607	0.04	0.05	0.05	0.05	1.00	1.00	1.00	0.14	0.14	1.00
FIG00955572: hypothetical protein;Note=FIG00955572: hypothetical protein	AFTH01000403.1: 100-1833	0.01	0.01	0.05	0.01	1.00	1.00	1.00	0.01	0.01	0.01
FIG00954449: hypothetical protein;Note=FIG00954449: hypothetical protein	AFTH01000756.1: 1215-3965	1.00	1.00	1.00	1.00	0.00	1.00	1.00	1.00	1.00	1.00
Two-component response regulator;Note=Two-component response regulator	AFTH01000398.1: 198-1703	1.00	1.00	1.00	1.00	1.00	1.00	1.00	1.00	1.00	0.00
FIG00962025: hypothetical protein;Note=FIG00962025: hypothetical protein	AFTH01000391.1: 668-829	1.00	1.00	1.00	1.00	1.00	1.00	1.00	1.00	1.00	0.00
Glutamate transport membrane-spanning protein;Note=Glutamate transport membrane-spanning protein	AFTH01000391.1: 1134-1799	1.00	1.00	1.00	1.00	1.00	1.00	1.00	1.00	1.00	0.00
Glutamate transport membrane-spanning protein;Note=Glutamate transport membrane-spanning protein	AFTH01000391.1: 1780-2439	1.00	1.00	1.00	1.00	1.00	1.00	1.00	1.00	1.00	0.00
ABC-type amino acid transport/signal transduction systems2C periplasmic component/domain;Note=ABC-type amino acid	AFTH01000391.1: 3194-3760	1.00	1.00	1.00	1.00	1.00	1.00	1.00	1.00	1.00	0.00
ABC-type amino acid transport/signal transduction systems2C periplasmic component/domain;Note=ABC-type amino acid	AFTH01000391.1: 3815-4066	1.00	1.00	1.00	1.00	1.00	1.00	1.00	1.00	1.00	0.00
transcriptional regulator2C AraC family protein;Note=transcriptional regulator2C AraC family protein	AFTH01000391.1: 5144-5989	1.00	1.00	1.00	1.00	1.00	1.00	1.00	1.00	1.00	0.00
candidate type III effector HolPtoQ;Note=candidate type III effector HolPtoQ	AFTH01000389.1: 443-1786	1.00	1.00	1.00	1.00	1.00	1.00	1.00	1.00	1.00	0.00
hypothetical protein;Note=hypothetical protein	AFTH01000389.1: 3226-3366	1.00	1.00	1.00	1.00	1.00	1.00	1.00	1.00	1.00	0.00
hypothetical protein;Note=hypothetical protein	AFTH01000389.1: 3317-3667	1.00	1.00	1.00	1.00	1.00	1.00	1.00	1.00	1.00	0.00
hypothetical protein;Note=hypothetical protein	AFTH01000387.1: 188-364	1.00	1.00	1.00	1.00	1.00	1.00	1.00	1.00	1.00	0.00
hypothetical protein;Note=hypothetical protein	AFTH01000376.1: 1752-1871	1.00	1.00	1.00	1.00	0.00	0.00	1.00	1.00	1.00	0.00
FIG00965780: hypothetical protein;Note=FIG00965780: hypothetical protein	AFTH01000372.1: 295-1002	1.00	1.00	1.00	1.00	1.00	1.00	1.00	1.00	1.00	0.00
type III effector AvrB4-2;Note=type III effector AvrB4-2	AFTH01000366.1: 182-1144	1.00	1.00	1.00	1.00	0.00	0.15	1.00	1.00	1.00	0.14
hypothetical protein;Note=hypothetical protein	AFTH01000366.1: 1672-1866	1.00	1.00	1.00	1.00	0.00	0.00	1.00	1.00	1.00	0.00

Supplementary Table 3. Comparison of gene content among *Psa* isolates based on aligning reads against the reference genome of *Psa* NCPPB3739 (GenBank:AFTH01000000).

TnpA transposase;Note=TnpA transposase	AFTH01000366.1: 1871-2008	1.00	1.00	1.00	1.00	0.00	0.00	1.00	1.00	1.00	0.60
Yersinia/Haemophilus virulence surface antigen family;Note=Yersinia/Haemophilus virulence surface antigen family	AFTH01000366.1: 2089-3162	1.00	1.00	1.00	1.00	0.00	0.09	1.00	1.00	1.00	1.00
Putative large exoprotein involved in heme utilization or adhesion of ShlA/HecA/FhaA family;Note=Putative large exoprot	AFTH01000365.1: 40-9012	0.99	0.98	0.99	0.99	0.00	1.00	1.00	1.00	0.99	0.99
NAD-dependent protein deacetylase of SIR2 family;Note=NAD-dependent protein deacetylase of SIR2 family	AFTH01000753.1: 334-552	1.00	1.00	1.00	1.00	1.00	1.00	1.00	1.00	1.00	0.00
Hemolysin activator protein precursor;Note=Hemolysin activator protein precursor	AFTH01000753.1: 1943-3523	1.00	1.00	1.00	1.00	1.00	1.00	1.00	1.00	1.00	0.05
Putative large exoprotein involved in heme utilization or adhesion of ShlA/HecA/FhaA family;Note=Putative large exoprot	AFTH01000753.1: 3548-12382	1.00	1.00	1.00	1.00	1.00	1.00	1.00	1.00	1.00	0.02
hypothetical protein;Note=hypothetical protein	AFTH01000753.1: 12939-13058	1.00	1.00	1.00	1.00	1.00	1.00	1.00	1.00	1.00	0.00
TnpA transposase;Note=TnpA transposase	AFTH01000752.1: 4486-4974	0.05	0.01	0.05	0.04	1.00	1.00	1.00	0.05	0.05	0.34
MFS general substrate transporter;Note=MFS general substrate transporter	AFTH01000330.1: 18627-19889	1.00	1.00	1.00	1.00	1.00	1.00	1.00	1.00	1.00	0.00
hypothetical protein;Note=hypothetical protein	AFTH01000330.1: 19912-20187	1.00	1.00	1.00	1.00	1.00	1.00	1.00	1.00	1.00	0.00
Dipeptide-binding ABC transporter2C periplasmic substrate-binding component (TC 3.A.1.5.2);Note=Dipeptide-binding AB	AFTH01000330.1: 21821-22039	1.00	1.00	1.00	1.00	1.00	1.00	1.00	1.00	1.00	0.00
Name=transposase transposase;Note=transposase	AFTH01000330.1: 22062-22286	0.36	0.35	0.36	0.35	1.00	1.00	1.00	0.37	0.37	0.00
NADH:flavin oxidoreductases2C Old Yellow Enzyme family;Note=NADH:flavin oxidoreductases2C Old Yellow Enzyme famil	AFTH01000319.1: 56-238	1.00	1.00	1.00	1.00	1.00	1.00	1.00	1.00	1.00	0.00
hypothetical protein;Note=hypothetical protein	AFTH01000319.1: 216-911	1.00	1.00	1.00	1.00	1.00	1.00	1.00	1.00	1.00	0.00
Permeases of the major facilitator superfamily;Note=Permeases of the major facilitator superfamily	AFTH01000319.1: 972-1358	1.00	1.00	1.00	1.00	1.00	1.00	1.00	1.00	1.00	0.00
NADH:flavin oxidoreductases2C Old Yellow Enzyme family;Note=NADH:flavin oxidoreductases2C Old Yellow Enzyme famil	AFTH01000319.1: 1416-2090	1.00	1.00	1.00	1.00	1.00	1.00	1.00	1.00	1.00	0.00
NADH:flavin oxidoreductases2C Old Yellow Enzyme family;Note=NADH:flavin oxidoreductases2C Old Yellow Enzyme famil	AFTH01000319.1: 2125-2526	0.65	0.64	0.65	0.65	1.00	1.00	1.00	0.66	0.66	0.00
Transcriptional regulator2C TetR family;Note=Transcriptional regulator2C TetR family	AFTH01000319.1: 2582-3163	0.99	1.00	1.00	1.00	1.00	1.00	1.00	1.00	1.00	0.00
hypothetical protein;Note=hypothetical protein	AFTH01000319.1: 4278-4457	1.00	1.00	1.00	1.00	1.00	1.00	1.00	1.00	1.00	0.00
transcriptional regulator2C AraC family;Note=transcriptional regulator2C AraC family	AFTH01000319.1: 4667-5650	1.00	1.00	1.00	1.00	1.00	1.00	1.00	1.00	1.00	0.01
hypothetical protein;Note=hypothetical protein	AFTH01000314.1: 2553-3047	1.00	1.00	1.00	1.00	1.00	1.00	1.00	1.00	1.00	0.03
Serine protease;Note=Serine protease	AFTH01000305.1: 327-2423	0.04	0.04	0.06	0.07	1.00	1.00	1.00	0.08	0.08	0.07
FIG00958716: hypothetical protein;Note=FIG00958716: hypothetical protein	AFTH01000305.1: 4304-4912	0.47	0.48	0.52	0.54	1.00	1.00	1.00	0.62	0.62	0.00
TnpA transposase;Note=TnpA transposase	AFTH01000301.1: 103-240	0.51	0.01	0.54	0.52	1.00	1.00	1.00	0.80	0.80	0.84
ParA family protein;Note=ParA family protein	AFTH01000301.1: 891-1172	1.00	1.00	1.00	1.00	1.00	1.00	1.00	1.00	1.00	0.00
ribose ABC transporter2C permease protein;Note=ribose ABC transporter2C permease protein	AFTH01000283.1: 234-815	1.00	1.00	1.00	1.00	1.00	1.00	1.00	1.00	1.00	0.00
Transketolase2C N-terminal section;Ontology_term=KEGG_ENZYME:2.2.1.1;Note=Transketolase2C N-terminal section;On	AFTH01000283.1: 1179-2015	1.00	0.98	1.00	1.00	1.00	1.00	1.00	1.00	1.00	0.00
Transketolase2C C-terminal section;Ontology_term=KEGG_ENZYME:2.2.1.1;Note=Transketolase2C C-terminal section;Ont	AFTH01000283.1: 2005-2937	1.00	1.00	1.00	1.00	1.00	1.00	1.00	1.00	1.00	0.05
Unknown pentose kinase TM0952;Note=Unknown pentose kinase TM0952	AFTH01000283.1: 2950-4443	0.93	0.96	0.92	0.93	1.00	1.00	1.00	0.95	0.95	0.01
hypothetical protein;Note=hypothetical protein	AFTH01000790.1: 608-724	1.00	1.00	1.00	1.00	1.00	1.00	1.00	1.00	1.00	0.00
FIG00635470: hypothetical protein;Note=FIG00635470: hypothetical protein	AFTH01000261.1: 16878-17774	1.00	1.00	1.00	1.00	1.00	1.00	1.00	1.00	1.00	0.00
Cellulose synthase operon protein C;Note=Cellulose synthase operon protein C	AFTH01000257.1: 288-4103	0.57	0.57	0.57	0.57	1.00	1.00	1.00	0.57	0.57	0.00
Colicin immunity protein;Note=Colicin immunity protein	AFTH01000253.1: 7515-7778	1.00	1.00	1.00	1.00	1.00	1.00	0.00	1.00	1.00	0.52
DNA integration/recombination/invertion protein;Note=DNA integration/recombination/invertion protein	AFTH01000244.1: 703-843	1.00	1.00	1.00	1.00	1.00	1.00	1.00	1.00	1.00	0.00
Name=Levansucrase;Ontology_term=KEGG_ENZYME:2.4.1.10;Note=Levansucrase Levansucrase;Ontology_term=KEGG_E	AFTH01000244.1: 6684-7979	1.00	1.00	1.00	1.00	1.00	1.00	1.00	1.00	1.00	0.00
hypothetical protein;Note=hypothetical protein	AFTH01000808.1: 186-344	1.00	1.00	1.00	1.00	0.00	0.00	1.00	1.00	1.00	0.00
TnpA transposase;Note=TnpA transposase	AFTH01000731.1: 377-1072	0.03	0.02	0.03	0.03	1.00	1.00	1.00	0.14	0.14	0.02
ATP-dependent helicase HrpB;Note=ATP-dependent helicase HrpB	AFTH01000217.1: 2220-4319	0.03	0.03	0.03	0.03	1.00	1.00	1.00	0.03	0.03	1.00
hypothetical protein;Note=hypothetical protein	AFTH01000190.1: 74-1408	0.74	0.73	0.73	0.73	1.00	1.00	1.00	0.74	0.74	0.00
hypothetical protein;Note=hypothetical protein	AFTH01000190.1: 1748-1864	1.00	1.00	1.00	1.00	1.00	1.00	1.00	1.00	1.00	0.00
hypothetical protein;Note=hypothetical protein	AFTH01000189.1: 248-508	1.00	1.00	1.00	1.00	1.00	1.00	1.00	1.00	1.00	0.00
hypothetical protein;Note=hypothetical protein	AFTH01000168.1: 283-528	1.00	1.00	1.00	1.00	1.00	1.00	1.00	1.00	1.00	0.00
site-specific recombinase2C phage integrase family;Note=site-specific recombinase2C phage integrase family	AFTH01000168.1: 525-1208	1.00	1.00	1.00	1.00	1.00	1.00	1.00	1.00	1.00	0.00
Unknown pentose utilization regulator 22C DeoR family;Note=Unknown pentose utilization regulator 22C DeoR family	AFTH01000163.1: 29-982	0.99	0.99	0.99	0.99	1.00	1.00	1.00	1.00	1.00	0.00
3-oxoacyl-[acyl-carrier protein] reductase;Ontology_term=KEGG_ENZYME:1.1.1.100;Note=3-oxoacyl-[acyl-carrier protein]	AFTH01000163.1: 1064-1864	1.00	0.99	1.00	1.00	1.00	1.00	1.00	1.00	1.00	0.00
Short chain dehydrogenase;Note=Short chain dehydrogenase	AFTH01000163.1: 1895-2659	1.00	1.00	1.00	1.00	1.00	1.00	1.00	1.00	1.00	0.00
D-xylose transport ATP-binding protein XylG;Note=D-xylose transport ATP-binding protein XylG	AFTH01000163.1: 2691-2837	1.00	1.00	1.00	1.00	1.00	1.00	1.00	1.00	1.00	0.00
ATP binding protein;Note=ATP binding protein	AFTH01000142.1: 204-1559	1.00	1.00	1.00	1.00	1.00	1.00	1.00	1.00	1.00	0.00
FIG00967211: hypothetical protein;Note=FIG00967211: hypothetical protein	AFTH01000142.1: 1709-2278	1.00	1.00	1.00	1.00	1.00	1.00	1.00	1.00	1.00	0.00
hypothetical protein;Note=hypothetical protein	AFTH01000132.1: 1455-2690	1.00	1.00	1.00	1.00	0.00	1.00	1.00	1.00	1.00	1.00
FIG00962378: hypothetical protein;Note=FIG00962378: hypothetical protein	AFTH01000724.1: 449-1399	1.00	1.00	1.00	1.00	1.00	1.00	1.00	1.00	1.00	0.00
hypothetical protein;Note=hypothetical protein	AFTH01000122.1: 1638-1781	1.00	1.00	1.00	1.00	0.00	1.00	1.00	1.00	1.00	1.00
hypothetical protein;Note=hypothetical protein	AFTH01000122.1: 1818-2363	1.00	1.00	1.00	1.00	0.00	1.00	1.00	1.00	1.00	1.00
NreA-like protein;Note=NreA-like protein	AFTH01000122.1: 2592-2885	1.00	1.00	1.00	1.00	0.00	1.00	1.00	1.00	1.00	1.00
hypothetical protein;Note=hypothetical protein	AFTH01000122.1: 3214-3342	1.00	1.00	1.00	1.00	0.00	1.00	1.00	1.00	1.00	1.00
COG1649 predicted glycoside hydrolase;Note=COG1649 predicted glycoside hydrolase	AFTH01000114.1: 425-1450	1.00	1.00	1.00	1.00	1.00	1.00	1.00	1.00	1.00	0.00
ABC-type amino acid transport2C signal transduction systems2C periplasmic component/domain;Note=ABC-type amino a	AFTH01000724.1: 12607-13362	0.03	0.03	0.04	0.04	1.00	1.00	1.00	0.04	0.04	1.00
FIG00961388: hypothetical protein;Note=FIG00961388: hypothetical protein	AFTH01000094.1: 1784-1903	1.00	1.00	1.00	1.00	1.00	1.00	1.00	1.00	1.00	0.00
FIG00961388: hypothetical protein;Note=FIG00961388: hypothetical protein	AFTH01000094.1: 2016-2360	1.00	1.00	1.00	1.00	1.00	1.00	1.00	1.00	1.00	0.00
hypothetical protein;Note=hypothetical protein	AFTH01000087.1: 7184-7303	1.00	1.00	1.00	1.00	1.00	1.00	1.00	1.00	1.00	0.00
conserved effector locus protein;Note=conserved effector locus protein	AFTH01000082.1: 532-1011	1.00	1.00	1.00	1.00	1.00	1.00	1.00	1.00	1.00	0.00
type III effector HopN1;Note=type III effector HopN1	AFTH01000082.1: 1025-2077	1.00	1.00	1.00	1.00	1.00	1.00	1.00	1.00	1.00	0.00
Transcriptional regulator2C LysR family;Note=Transcriptional regulator2C LysR family	AFTH01000077.1: 800-1699	1.00	1.00	1.00	1.00	1.00	1.00	1.00	1.00	1.00	0.00

Supplementary Table 3. Comparison of gene content among *Psa* isolates based on aligning reads against the reference genome of *Psa* NCPPB3739 (GenBank:AFTH01000000).

hypothetical protein;Note=hypothetical protein	AFTH01000070.1: 36-854	1.00	1.00	1.00	1.00	1.00	1.00	1.00	1.00	1.00	0.00
hypothetical protein;Note=hypothetical protein	AFTH01000808.1: 466-867	1.00	1.00	1.00	1.00	0.00	0.00	1.00	1.00	1.00	0.00
Xylose ABC transporter2C periplasmic xylose-binding protein XylF;Note=Xylose ABC transporter2C periplasmic xylose-bind	AFTH01000050.1: 1402-2334	0.99	0.96	1.00	1.00	1.00	1.00	1.00	1.00	1.00	0.00
ribose ABC transporter2C permease protein;Note=ribose ABC transporter2C permease protein	AFTH01000050.1: 2401-2604	1.00	0.93	1.00	1.00	1.00	1.00	1.00	1.00	1.00	0.00
YoeB toxin protein;Note=YoeB toxin protein	AFTH01000047.1: 13047-13349	1.00	1.00	1.00	1.00	1.00	1.00	1.00	1.00	1.00	0.00
YefM protein (antitoxin to YoeB);Note=YefM protein (antitoxin to YoeB)	AFTH01000047.1: 13321-13599	1.00	1.00	1.00	1.00	1.00	1.00	1.00	1.00	1.00	0.00
Dipeptide-binding ABC transporter2C periplasmic substrate-binding component (TC 3.A.1.5.2);Note=Dipeptide-binding AB	AFTH01000046.1: 560-2137	1.00	1.00	1.00	1.00	1.00	1.00	1.00	1.00	1.00	0.00
Dipeptide transport system permease protein DppB (TC 3.A.1.5.2)%3B putative hemin permease;Note=Dipeptide transpo	AFTH01000046.1: 2158-3168	1.00	1.00	1.00	1.00	1.00	1.00	1.00	1.00	1.00	0.00
Dipeptide transport system permease protein DppC (TC 3.A.1.5.2);Note=Dipeptide transport system permease protein Dp	AFTH01000046.1: 3165-4016	1.00	1.00	1.00	1.00	1.00	1.00	1.00	1.00	1.00	0.00
Dipeptide transport ATP-binding protein DppD (TC 3.A.1.5.2);Note=Dipeptide transport ATP-binding protein DppD (TC 3.A	AFTH01000046.1: 4009-4857	1.00	1.00	1.00	1.00	1.00	1.00	1.00	1.00	1.00	0.00
dipeptide ABC transporter2C ATP binding protein;Note=dipeptide ABC transporter2C ATP binding protein	AFTH01000046.1: 4854-5567	1.00	1.00	1.00	1.00	1.00	1.00	1.00	1.00	1.00	0.00
Cytochrome B561;Note=Cytochrome B561	AFTH01000046.1: 5672-6229	1.00	1.00	1.00	1.00	1.00	1.00	1.00	1.00	1.00	0.00
transmembrane sensor2C putative;Note=transmembrane sensor2C putative	AFTH01000046.1: 8364-9329	1.00	1.00	1.00	1.00	1.00	1.00	1.00	1.00	1.00	0.00
TonB-dependent ferric achromobactin receptor protein;Note=TonB-dependent ferric achromobactin receptor protein	AFTH01000046.1: 9399-11801	1.00	1.00	1.00	1.00	1.00	1.00	1.00	1.00	1.00	0.00
hypothetical protein;Note=hypothetical protein	AFTH01000044.1: 1288-1479	0.28	0.27	0.28	0.28	1.00	1.00	1.00	0.67	0.67	0.00
VgrG protein;Note=VgrG protein	AFTH01000032.1: 1558-3585	1.00	1.00	1.00	1.00	1.00	1.00	1.00	1.00	1.00	0.04
VgrG protein;Note=VgrG protein	AFTH01000032.1: 4430-7744	1.00	1.00	1.00	1.00	1.00	1.00	1.00	1.00	1.00	0.02
Glucans biosynthesis protein D precursor;Note=Glucans biosynthesis protein D precursor	AFTH01000008.1: 34-351	1.00	1.00	1.00	1.00	1.00	1.00	1.00	1.00	1.00	0.00
hypothetical protein;Note=hypothetical protein	AFTH01000719.1: 13957-14289	1.00	1.00	1.00	1.00	1.00	1.00	0.00	1.00	1.00	1.00
avirulence protein;Note=avirulence protein	AFTH01000808.1: 1552-2571	1.00	1.00	1.00	1.00	0.00	0.08	1.00	1.00	1.00	0.00
hypothetical protein;Note=hypothetical protein	AFTH01000710.1: 10142-10405	1.00	1.00	1.00	1.00	1.00	1.00	1.00	1.00	1.00	0.00
RecA/RadA recombinase;Note=RecA/RadA recombinase	AFTH01000783.1: 116-421	1.00	1.00	1.00	1.00	0.00	1.00	1.00	1.00	1.00	1.00
DNA polymerase-like protein PA0670;Note=DNA polymerase-like protein PA0670	AFTH01000783.1: 429-1844	1.00	1.00	1.00	1.00	0.00	1.00	1.00	1.00	1.00	1.00
FIG00958014: hypothetical protein;Note=FIG00958014: hypothetical protein	AFTH01000707.1: 15984-16247	1.00	1.00	1.00	1.00	1.00	1.00	1.00	1.00	1.00	0.00
DNA polymerase III alpha subunit;Ontology_term=KEGG_ENZYME:2.7.7.7;Note=DNA polymerase III alpha subunit;Ontolog	AFTH01000783.1: 1841-2605	1.00	1.00	1.00	1.00	0.00	1.00	1.00	1.00	1.00	0.99
Error-prone2C lesion bypass DNA polymerase V (UmuC);Note=Error-prone2C lesion bypass DNA polymerase V (UmuC)	AFTH01000705.1: 3593-3841	0.01	0.00	0.02	0.02	1.00	1.00	1.00	0.31	0.31	0.00
hypothetical protein;Note=hypothetical protein	AFTH01000780.1: 76-198	1.00	1.00	1.00	1.00	1.00	1.00	1.00	1.00	1.00	0.01
SOS-response repressor and protease LexA;Ontology_term=KEGG_ENZYME:3.4.21.88;Note=SOS-response repressor and p	AFTH01000688.1: 31-648	1.00	1.00	1.00	1.00	0.00	1.00	1.00	1.00	1.00	1.00
Uncharacterized protein ImpA;Note=Uncharacterized protein ImpA	AFTH01000681.1: 158-1489	0.04	0.04	0.05	0.04	1.00	1.00	1.00	0.10	0.10	1.00
PUTATIVE AVRPPHE AVIRULENCE PROTEIN-HOMOLOG;Note=PUTATIVE AVRPPHE AVIRULENCE PROTEIN-HOMOLOG	**AFTH01000677.1: 57-1061**	**1.00**	**1.00**	**1.00**	**1.00**	**0.00**	**0.22**	**1.00**	**1.00**	**1.00**	**0.00**
hypothetical protein;Note=hypothetical protein	AFTH01000645.1: 1090-1263	0.59	0.58	0.59	0.60	1.00	1.00	1.00	0.59	0.59	0.00
FIG004780: hypothetical protein in PFGI-1-like cluster;Note=FIG004780: hypothetical protein in PFGI-1-like cluster	AFTH01000635.1: 1557-2294	0.05	0.06	0.07	0.07	1.00	1.00	1.00	0.96	0.96	0.00
Protein with ParB-like nuclease domain in PFGI-1-like cluster;Note=Protein with ParB-like nuclease domain in PFGI-1-like c	AFTH01000635.1: 2331-3632	0.05	0.05	0.05	0.05	1.00	1.00	1.00	0.86	0.86	0.03
TnpA transposase;Note=TnpA transposase	AFTH01000622.1: 2076-4409	0.02	0.02	0.05	0.02	0.91	1.00	0.43	0.05	0.05	0.02
