# Supplementary material for: Pseudomonas syringae pv. actinidiae (PSA) Isolates from Recent Bacterial Canker of Kiwifruit Outbreaks Belong to the Same Genetic Lineage
Source: PLoS One. 2012 May 9;7(5):e36518. doi: 10.1371/journal.pone.0036518 (PMC3348921; doi:10.1371/journal.pone.0036518)
Supplement: Table S4 — Comparison of gene content among PSA isolates based on aligning reads against the reference genome of Psa NCPPB3871 (GenBank:AFTF01000000). (PDF) [file pone.0036518.s004.pdf]

**Supplementary Table 4.** Comparison of gene content among *Psa* isolates based on aligning reads against the reference genome of *Psa* NCPPB3871 (GenBank:AFTF01000000).

| Gene | Genomic coordinates | 346 | CFBP7285 | CFBP7286 | CFBP7287 | PsaKN.2 | Kw41 | PA459 | CH2010-5 | CH2010-6 | NCPPB2598 |
|---|---|---|---|---|---|---|---|---|---|---|---|
| As predicted by RAST (http://http://rast.nmpdr.org/) | (GenBank accession: start-end) | Portugal | Italy | Italy | Italy | Korea | Japan | Japan | China | China | Japan (theae) |
| site-specific recombinase2C phage integrase family;Note=site-specific recombinase2C phage integrase family | AFTF01000284.1: 1160-2170 | 1.00 | 1.00 | 1.00 | 1.00 | 1.00 | 1.00 | 1.00 | 1.00 | 1.00 | 0.00 |
| Serine protease;Note=Serine protease | AFTF01000284.1: 2148-3203 | 0.08 | 0.07 | 0.08 | 0.08 | 1.00 | 1.00 | 1.00 | 0.08 | 0.08 | 0.00 |
| Long-chain-fatty-acid--CoA ligase;Ontology_term=KEGG_ENZYME:6.2.1.3;Note=Long-chain-fatty-acid--CoA ligas | AFTF01000280.1: 1865-4321 | 0.01 | 0.01 | 0.01 | 0.01 | 1.00 | 1.00 | 1.00 | 0.01 | 0.01 | 0.01 |
| Name=titin titin;Note=titin | AFTF01000278.1: 882-2111 | 1.00 | 1.00 | 1.00 | 1.00 | 0.00 | 1.00 | 1.00 | 1.00 | 1.00 | 1.00 |
| DNA polymerase III alpha subunit;Ontology_term=KEGG_ENZYME:2.7.7.7;Note=DNA polymerase III alpha subu | AFTF01000278.1: 2169-2933 | 1.00 | 1.00 | 1.00 | 1.00 | 0.00 | 1.00 | 1.00 | 1.00 | 1.00 | 0.99 |
| DNA polymerase-like protein PA0670;Note=DNA polymerase-like protein PA0670 | AFTF01000278.1: 2930-4345 | 1.00 | 1.00 | 1.00 | 1.00 | 0.00 | 1.00 | 1.00 | 1.00 | 1.00 | 1.00 |
| RecA/RadA recombinase;Note=RecA/RadA recombinase | AFTF01000278.1: 4353-4976 | 1.00 | 1.00 | 1.00 | 1.00 | 0.00 | 1.00 | 1.00 | 1.00 | 1.00 | 1.00 |
| SOS-response repressor and protease LexA;Ontology_term=KEGG_ENZYME:3.4.21.88;Note=SOS-response repr | AFTF01000278.1: 4973-5590 | 1.00 | 1.00 | 1.00 | 1.00 | 0.00 | 1.00 | 1.00 | 1.00 | 1.00 | 1.00 |
| FIG00961388: hypothetical protein;Note=FIG00961388: hypothetical protein | AFTF01000275.1: 1861-1980 | 1.00 | 1.00 | 1.00 | 1.00 | 1.00 | 1.00 | 1.00 | 1.00 | 1.00 | 0.00 |
| FIG00961388: hypothetical protein;Note=FIG00961388: hypothetical protein | AFTF01000275.1: 2093-2437 | 1.00 | 1.00 | 1.00 | 1.00 | 1.00 | 1.00 | 1.00 | 1.00 | 1.00 | 0.00 |
| hypothetical protein;Note=hypothetical protein | AFTF01000432.1: 1709-2545 | 1.00 | 1.00 | 1.00 | 1.00 | 1.00 | 1.00 | 1.00 | 1.00 | 1.00 | 0.00 |
| membrane protein2C putative;Note=membrane protein2C putative | AFTF01000432.1: 2671-3198 | 1.00 | 1.00 | 1.00 | 1.00 | 1.00 | 1.00 | 1.00 | 1.00 | 1.00 | 0.00 |
| hypothetical protein;Note=hypothetical protein | AFTF01000263.1: 1639-1782 | 1.00 | 1.00 | 1.00 | 1.00 | 0.00 | 1.00 | 1.00 | 1.00 | 1.00 | 1.00 |
| hypothetical protein;Note=hypothetical protein | AFTF01000263.1: 1894-2364 | 1.00 | 1.00 | 1.00 | 1.00 | 0.00 | 1.00 | 1.00 | 1.00 | 1.00 | 1.00 |
| NreA-like protein;Note=NreA-like protein | AFTF01000263.1: 2593-2886 | 1.00 | 1.00 | 1.00 | 1.00 | 0.00 | 1.00 | 1.00 | 1.00 | 1.00 | 1.00 |
| Nickel and cobalt efflux transporter rcnA;Note=Nickel and cobalt efflux transporter rcnA | AFTF01000263.1: 2894-3766 | 1.00 | 1.00 | 1.00 | 1.00 | 0.00 | 1.00 | 1.00 | 1.00 | 1.00 | 1.00 |
| Protein with ParB-like nuclease domain in PFGI-1-like cluster;Note=Protein with ParB-like nuclease domain in P | AFTF01000262.1: 4586-5038 | 0.10 | 0.13 | 0.13 | 0.14 | 1.00 | 1.00 | 1.00 | 0.53 | 0.53 | 0.00 |
| Name=ORF2 ORF2;Note=ORF2 | AFTF01000450.1: 58-1182 | 1.00 | 1.00 | 1.00 | 1.00 | 0.00 | 0.19 | 1.00 | 1.00 | 1.00 | 0.00 |
| Protein with ParB-like nuclease domain in PFGI-1-like cluster;Note=Protein with ParB-like nuclease domain in P | AFTF01000259.1: 58-1359 | 0.05 | 0.05 | 0.05 | 0.05 | 1.00 | 1.00 | 1.00 | 0.86 | 0.86 | 0.03 |
| FIG004780: hypothetical protein in PFGI-1-like cluster;Note=FIG004780: hypothetical protein in PFGI-1-like clus | AFTF01000259.1: 1396-2133 | 0.05 | 0.06 | 0.07 | 0.07 | 1.00 | 1.00 | 1.00 | 0.96 | 0.96 | 0.00 |
| DNA/RNA helicase in PFGI-1-like cluster;Note=DNA/RNA helicase in PFGI-1-like cluster | AFTF01000259.1: 8682-10466 | 0.04 | 0.04 | 0.05 | 0.05 | 1.00 | 1.00 | 1.00 | 0.91 | 0.90 | 0.79 |
| Conjugative transfer protein PilL in PFGI-1-like cluster;Note=Conjugative transfer protein PilL in PFGI-1-like clust | AFTF01000259.1: 12010-13155 | 0.03 | 0.04 | 0.04 | 0.17 | 1.00 | 1.00 | 1.00 | 0.34 | 0.28 | 1.00 |
| ABC-type amino acid transport2C signal transduction systems2C periplasmic component/domain;Note=ABC-typ | AFTF01000258.1: 1775-2530 | 0.03 | 0.03 | 0.04 | 0.04 | 1.00 | 1.00 | 1.00 | 0.04 | 0.04 | 1.00 |
| FIG00962378: hypothetical protein;Note=FIG00962378: hypothetical protein | AFTF01000258.1: 13738-14688 | 1.00 | 1.00 | 1.00 | 1.00 | 1.00 | 1.00 | 1.00 | 1.00 | 1.00 | 0.00 |
| hypothetical protein;Note=hypothetical protein | AFTF01000448.1: 2842-2976 | 1.00 | 1.00 | 1.00 | 1.00 | 1.00 | 1.00 | 1.00 | 1.00 | 1.00 | 0.00 |
| TnpA transposase;Note=TnpA transposase | AFTF01000240.1: 3415-4110 | 0.03 | 0.02 | 0.03 | 0.03 | 1.00 | 1.00 | 1.00 | 0.14 | 0.14 | 0.02 |
| DNA integration/recombination/invertion protein;Note=DNA integration/recombination/invertion protein | AFTF01000448.1: 3018-3158 | 1.00 | 1.00 | 1.00 | 1.00 | 1.00 | 1.00 | 1.00 | 1.00 | 1.00 | 0.00 |
| FIG00960122: hypothetical protein;Note=FIG00960122: hypothetical protein | AFTF01000230.1: 718-2076 | 0.93 | 0.94 | 0.94 | 0.95 | 1.00 | 1.00 | 1.00 | 0.96 | 0.96 | 0.00 |
| Name=Integrase Integrase;Note=Integrase | AFTF01000230.1: 2348-3583 | 0.05 | 0.05 | 0.04 | 0.05 | 1.00 | 1.00 | 1.00 | 0.11 | 0.11 | 0.00 |
| Serine protease;Note=Serine protease | AFTF01000226.1: 414-2510 | 0.04 | 0.04 | 0.06 | 0.07 | 1.00 | 1.00 | 1.00 | 0.08 | 0.08 | 0.07 |
| FIG00958716: hypothetical protein;Note=FIG00958716: hypothetical protein | AFTF01000226.1: 4391-4999 | 0.47 | 0.48 | 0.52 | 0.54 | 1.00 | 1.00 | 1.00 | 0.62 | 0.62 | 0.00 |
| TonB-dependent receptor%3B Outer membrane receptor for ferrienterochelin and colicins;Note=TonB-depend | AFTF01000225.1: 51-2450 | 0.02 | 0.02 | 0.02 | 0.02 | 1.00 | 1.00 | 1.00 | 0.09 | 0.09 | 0.02 |
| Methyl-accepting chemotaxis protein;Note=Methyl-accepting chemotaxis protein | AFTF01000223.1: 3245-3619 | 0.82 | 0.96 | 1.00 | 0.97 | 0.00 | 0.00 | 1.00 | 1.00 | 1.00 | 1.00 |
| hypothetical protein;Note=hypothetical protein | AFTF01000223.1: 3677-5083 | 0.98 | 0.98 | 1.00 | 1.00 | 0.00 | 0.00 | 1.00 | 1.00 | 1.00 | 1.00 |
| Aconitase B;Note=Aconitase B | AFTF01000223.1: 5093-5422 | 1.00 | 1.00 | 1.00 | 1.00 | 0.00 | 0.16 | 1.00 | 1.00 | 1.00 | 1.00 |
| ATP-dependent helicase HrpB;Note=ATP-dependent helicase HrpB | AFTF01000222.1: 2218-4317 | 0.03 | 0.03 | 0.03 | 0.03 | 1.00 | 1.00 | 1.00 | 0.03 | 0.03 | 1.00 |
| hypothetical protein;Note=hypothetical protein | AFTF01000218.1: 585-701 | 1.00 | 1.00 | 1.00 | 1.00 | 1.00 | 1.00 | 1.00 | 1.00 | 1.00 | 0.00 |
| hypothetical protein;Note=hypothetical protein | AFTF01000218.1: 1041-3584 | 0.86 | 0.86 | 0.86 | 0.86 | 1.00 | 1.00 | 1.00 | 0.86 | 0.86 | 0.00 |
| Protein involved in meta-pathway of phenol degradation;Note=Protein involved in meta-pathway of phenol de | AFTF01000216.1: 47276-48169 | 0.04 | 0.04 | 0.05 | 0.05 | 1.00 | 1.00 | 1.00 | 0.05 | 0.05 | 0.86 |
| FIG045374: Type II restriction enzyme2C methylase subunit YeeA;Note=FIG045374: Type II restriction enzyme2 | AFTF01000216.1: 48154-50226 | 1.00 | 1.00 | 1.00 | 1.00 | 1.00 | 1.00 | 1.00 | 1.00 | 1.00 | 0.00 |
| hypothetical protein;Note=hypothetical protein | AFTF01000216.1: 51011-51142 | 1.00 | 1.00 | 1.00 | 1.00 | 1.00 | 1.00 | 1.00 | 1.00 | 1.00 | 0.00 |
| hypothetical protein;Note=hypothetical protein | AFTF01000211.1: 1067-1186 | 1.00 | 1.00 | 1.00 | 1.00 | 1.00 | 1.00 | 1.00 | 1.00 | 1.00 | 0.00 |
| TnpA transposase;Note=TnpA transposase | AFTF01000210.1: 4484-4972 | 0.05 | 0.05 | 0.05 | 0.05 | 1.00 | 1.00 | 1.00 | 0.05 | 0.05 | 0.32 |
| hypothetical protein;Note=hypothetical protein | AFTF01000206.1: 10136-10399 | 1.00 | 1.00 | 1.00 | 1.00 | 1.00 | 1.00 | 1.00 | 1.00 | 1.00 | 0.00 |
| Putative heat shock protein YegD;Note=Putative heat shock protein YegD | AFTF01000198.1: 26591-28156 | 1.00 | 1.00 | 1.00 | 1.00 | 1.00 | 1.00 | 1.00 | 1.00 | 1.00 | 0.00 |
| hypothetical protein;Note=hypothetical protein | AFTF01000198.1: 28706-28870 | 1.00 | 1.00 | 1.00 | 1.00 | 1.00 | 1.00 | 0.90 | 1.00 | 1.00 | 0.00 |
| Unknown pentose utilization regulator 22C DeoR family;Note=Unknown pentose utilization regulator 22C DeoR | AFTF01000189.1: 30-983 | 0.99 | 0.99 | 0.99 | 0.99 | 1.00 | 1.00 | 1.00 | 1.00 | 1.00 | 0.00 |
| 3-oxoacyl-[acyl-carrier protein] reductase;Ontology_term=KEGG_ENZYME:1.1.1.100;Note=3-oxoacyl-[acyl-carri | AFTF01000189.1: 1065-1865 | 1.00 | 0.99 | 1.00 | 1.00 | 1.00 | 1.00 | 1.00 | 1.00 | 1.00 | 0.00 |
| Short chain dehydrogenase;Note=Short chain dehydrogenase | AFTF01000189.1: 1896-2660 | 1.00 | 1.00 | 1.00 | 1.00 | 1.00 | 1.00 | 1.00 | 1.00 | 1.00 | 0.00 |
| D-xylose transport ATP-binding protein XylG;Note=D-xylose transport ATP-binding protein XylG | AFTF01000189.1: 2692-4221 | 1.00 | 0.99 | 1.00 | 1.00 | 1.00 | 1.00 | 1.00 | 1.00 | 1.00 | 0.00 |
| Xylose ABC transporter2C periplasmic xylose-binding protein XylF;Note=Xylose ABC transporter2C periplasmic x | AFTF01000189.1: 4237-5169 | 0.99 | 0.96 | 1.00 | 1.00 | 1.00 | 1.00 | 1.00 | 1.00 | 1.00 | 0.00 |
| ribose ABC transporter2C permease protein;Note=ribose ABC transporter2C permease protein | AFTF01000189.1: 5236-5658 | 1.00 | 1.00 | 1.00 | 1.00 | 1.00 | 1.00 | 1.00 | 1.00 | 1.00 | 0.00 |
| ribose ABC transporter2C permease protein;Note=ribose ABC transporter2C permease protein | AFTF01000189.1: 5659-6240 | 1.00 | 1.00 | 1.00 | 1.00 | 1.00 | 1.00 | 1.00 | 1.00 | 1.00 | 0.00 |
| Transketolase2C N-terminal section;Ontology_term=KEGG_ENZYME:2.2.1.1;Note=Transketolase2C N-terminal s | AFTF01000189.1: 6604-7440 | 1.00 | 0.98 | 1.00 | 1.00 | 1.00 | 1.00 | 1.00 | 1.00 | 1.00 | 0.00 |
| Transketolase2C C-terminal section;Ontology_term=KEGG_ENZYME:2.2.1.1;Note=Transketolase2C C-terminal s | AFTF01000189.1: 7430-8362 | 1.00 | 1.00 | 1.00 | 1.00 | 1.00 | 1.00 | 1.00 | 1.00 | 1.00 | 0.05 |
| Unknown pentose kinase TM0952;Note=Unknown pentose kinase TM0952 | AFTF01000189.1: 8375-9868 | 0.93 | 0.96 | 0.92 | 0.93 | 1.00 | 1.00 | 1.00 | 0.95 | 0.95 | 0.01 |
| Glucans biosynthesis protein D precursor;Note=Glucans biosynthesis protein D precursor | AFTF01000187.1: 22766-23083 | 1.00 | 1.00 | 1.00 | 1.00 | 1.00 | 1.00 | 1.00 | 1.00 | 1.00 | 0.00 |
| putative integrase protein;Note=putative integrase protein | AFTF01000444.1: 482-2140 | 1.00 | 1.00 | 1.00 | 1.00 | 0.00 | 0.14 | 1.00 | 1.00 | 1.00 | 1.00 |
| avirulence protein;Note=avirulence protein | AFTF01000184.1: 16557-17003 | 1.00 | 1.00 | 1.00 | 1.00 | 1.00 | 1.00 | 1.00 | 1.00 | 1.00 | 0.00 |
| hypothetical protein;Note=hypothetical protein | AFTF01000444.1: 2207-2347 | 1.00 | 1.00 | 1.00 | 1.00 | 0.00 | 0.43 | 1.00 | 1.00 | 1.00 | 1.00 |
| FIG00635470: hypothetical protein;Note=FIG00635470: hypothetical protein | AFTF01000178.1: 21173-22069 | 1.00 | 1.00 | 1.00 | 1.00 | 1.00 | 1.00 | 1.00 | 1.00 | 1.00 | 0.00 |

| | | | | | | | | | | | |
|---|---|---|---|---|---|---|---|---|---|---|---|
| FIG00962025: hypothetical protein;Note=FIG00962025: hypothetical protein | AFTF01000174.1: 669-830 | 1.00 | 1.00 | 1.00 | 1.00 | 1.00 | 1.00 | 1.00 | 1.00 | 1.00 | 0.00 |
| Glutamate transport membrane-spanning protein;Note=Glutamate transport membrane-spanning protein | AFTF01000174.1: 1135-1800 | 1.00 | 1.00 | 1.00 | 1.00 | 1.00 | 1.00 | 1.00 | 1.00 | 1.00 | 0.00 |
| Glutamate transport membrane-spanning protein;Note=Glutamate transport membrane-spanning protein | AFTF01000174.1: 1781-2440 | 1.00 | 1.00 | 1.00 | 1.00 | 1.00 | 1.00 | 1.00 | 1.00 | 1.00 | 0.00 |
| ABC-type amino acid transport/signal transduction systems2C periplasmic component/domain;Note=ABC-type | AFTF01000174.1: 3195-3761 | 1.00 | 1.00 | 1.00 | 1.00 | 1.00 | 1.00 | 1.00 | 1.00 | 1.00 | 0.00 |
| ABC-type amino acid transport/signal transduction systems2C periplasmic component/domain;Note=ABC-type | AFTF01000174.1: 3816-4067 | 1.00 | 1.00 | 1.00 | 1.00 | 1.00 | 1.00 | 1.00 | 1.00 | 1.00 | 0.00 |
| NADH:flavin oxidoreductases2C Old Yellow Enzyme family;Note=NADH:flavin oxidoreductases2C Old Yellow En | AFTF01000174.1: 5172-6269 | 1.00 | 1.00 | 1.00 | 1.00 | 1.00 | 1.00 | 1.00 | 1.00 | 1.00 | 0.00 |
| hypothetical protein;Note=hypothetical protein | AFTF01000174.1: 6247-6942 | 1.00 | 1.00 | 1.00 | 1.00 | 1.00 | 1.00 | 1.00 | 1.00 | 1.00 | 0.00 |
| Permeases of the major facilitator superfamily;Note=Permeases of the major facilitator superfamily | AFTF01000174.1: 7003-7389 | 1.00 | 1.00 | 1.00 | 1.00 | 1.00 | 1.00 | 1.00 | 1.00 | 1.00 | 0.00 |
| NADH:flavin oxidoreductases2C Old Yellow Enzyme family;Note=NADH:flavin oxidoreductases2C Old Yellow En | AFTF01000174.1: 7447-8115 | 1.00 | 1.00 | 1.00 | 1.00 | 1.00 | 1.00 | 1.00 | 1.00 | 1.00 | 0.00 |
| NADH:flavin oxidoreductases2C Old Yellow Enzyme family;Note=NADH:flavin oxidoreductases2C Old Yellow En | AFTF01000174.1: 8156-8557 | 0.65 | 0.64 | 0.65 | 0.65 | 1.00 | 1.00 | 1.00 | 0.66 | 0.66 | 0.00 |
| Transcriptional regulator2C TetR family;Note=Transcriptional regulator2C TetR family | AFTF01000174.1: 8613-9194 | 0.99 | 1.00 | 1.00 | 1.00 | 1.00 | 1.00 | 1.00 | 1.00 | 1.00 | 0.00 |
| hypothetical protein;Note=hypothetical protein | AFTF01000174.1: 10309-10488 | 1.00 | 1.00 | 1.00 | 1.00 | 1.00 | 1.00 | 1.00 | 1.00 | 1.00 | 0.00 |
| transcriptional regulator2C AraC family;Note=transcriptional regulator2C AraC family | AFTF01000174.1: 10698-11681 | 1.00 | 1.00 | 1.00 | 1.00 | 1.00 | 1.00 | 1.00 | 1.00 | 1.00 | 0.01 |
| ABC-type polar amino acid transport system2C ATPase component;Note=ABC-type polar amino acid transport s | AFTF01000169.1: 50794-51591 | 1.00 | 1.00 | 1.00 | 1.00 | 1.00 | 1.00 | 1.00 | 1.00 | 1.00 | 0.01 |
| ABC-type amino acid transport/signal transduction systems2C periplasmic component/domain;Note=ABC-type | AFTF01000169.1: 52973-53326 | 1.00 | 1.00 | 1.00 | 1.00 | 1.00 | 1.00 | 1.00 | 1.00 | 1.00 | 0.00 |
| ABC-type amino acid transport/signal transduction systems2C periplasmic component/domain;Note=ABC-type | AFTF01000169.1: 53545-53844 | 1.00 | 1.00 | 1.00 | 1.00 | 1.00 | 1.00 | 1.00 | 1.00 | 1.00 | 0.00 |
| avirulence protein;Note=avirulence protein | AFTF01000442.1: 1060-2079 | 1.00 | 1.00 | 1.00 | 1.00 | 0.00 | 0.08 | 1.00 | 1.00 | 1.00 | 0.00 |
| hypothetical protein;Note=hypothetical protein | AFTF01000442.1: 2764-2886 | 1.00 | 1.00 | 1.00 | 1.00 | 0.00 | 0.00 | 1.00 | 1.00 | 1.00 | 0.00 |
| hypothetical protein;Note=hypothetical protein | AFTF01000442.1: 3287-3445 | 1.00 | 1.00 | 1.00 | 1.00 | 0.00 | 0.00 | 1.00 | 1.00 | 1.00 | 0.00 |
| Putative large exoprotein involved in heme utilization or adhesion of ShlA/HecA/FhaA family;Note=Putative larg | AFTF01000159.1: 28-11799 | 0.99 | 0.99 | 0.99 | 0.99 | 0.00 | 1.00 | 1.00 | 1.00 | 1.00 | 0.99 |
| Cellulose synthase operon protein C;Note=Cellulose synthase operon protein C | AFTF01000158.1: 38043-41858 | 0.57 | 0.57 | 0.57 | 0.57 | 1.00 | 1.00 | 1.00 | 0.57 | 0.57 | 0.00 |
| Name=transposase transposase;Note=transposase | AFTF01000442.1: 3435-3845 | 1.00 | 1.00 | 1.00 | 1.00 | 0.00 | 0.00 | 1.00 | 1.00 | 1.00 | 0.00 |
| YoeB toxin protein;Note=YoeB toxin protein | AFTF01000154.1: 15016-15318 | 1.00 | 1.00 | 1.00 | 1.00 | 1.00 | 1.00 | 1.00 | 1.00 | 1.00 | 0.00 |
| Endoglucanase precursor;Ontology_term=KEGG_ENZYME:3.2.1.4;Note=Endoglucanase precursor;Ontology_ter | AFTF01000137.1: 44172-45206 | 1.00 | 1.00 | 1.00 | 1.00 | 1.00 | 1.00 | 1.00 | 1.00 | 1.00 | 0.05 |
| Transposase and inactivated derivatives;Note=Transposase and inactivated derivatives | AFTF01000128.1: 74-1123 | 0.03 | 0.03 | 0.03 | 0.03 | 1.00 | 1.00 | 0.03 | 0.03 | 0.03 | 0.03 |
| FIG00965780: hypothetical protein;Note=FIG00965780: hypothetical protein | AFTF01000085.1: 19-573 | 1.00 | 1.00 | 1.00 | 1.00 | 1.00 | 1.00 | 1.00 | 1.00 | 1.00 | 0.00 |
| FIG00965780: hypothetical protein;Note=FIG00965780: hypothetical protein | AFTF01000077.1: 195-1148 | 1.00 | 1.00 | 1.00 | 1.00 | 1.00 | 1.00 | 1.00 | 1.00 | 1.00 | 0.00 |
| VgrG protein;Note=VgrG protein | AFTF01000077.1: 1112-4426 | 1.00 | 1.00 | 1.00 | 1.00 | 1.00 | 1.00 | 1.00 | 1.00 | 1.00 | 0.02 |
| VgrG protein;Note=VgrG protein | AFTF01000077.1: 5271-7298 | 1.00 | 1.00 | 1.00 | 1.00 | 1.00 | 1.00 | 1.00 | 1.00 | 1.00 | 0.04 |
| Yersinia/Haemophilus virulence surface antigen family;Note=Yersinia/Haemophilus virulence surface antigen fa | AFTF01000074.1: 446-1519 | 1.00 | 1.00 | 1.00 | 1.00 | 0.00 | 0.09 | 1.00 | 1.00 | 1.00 | 1.00 |
| hypothetical protein;Note=hypothetical protein | AFTF01000074.1: 1742-1936 | 1.00 | 1.00 | 1.00 | 1.00 | 0.00 | 0.00 | 1.00 | 1.00 | 1.00 | 0.00 |
| type III effector AvrB4-2;Note=type III effector AvrB4-2 | AFTF01000074.1: 2464-3426 | 1.00 | 1.00 | 1.00 | 1.00 | 0.00 | 0.15 | 1.00 | 1.00 | 1.00 | 0.14 |
| Name=transposase transposase;Note=transposase | AFTF01000072.1: 324-548 | 0.36 | 0.35 | 0.36 | 0.35 | 1.00 | 1.00 | 1.00 | 0.37 | 0.37 | 0.00 |
| Dipeptide-binding ABC transporter2C periplasmic substrate-binding component (TC 3.A.1.5.2);Note=Dipeptide- | AFTF01000072.1: 571-789 | 1.00 | 1.00 | 1.00 | 1.00 | 1.00 | 1.00 | 1.00 | 1.00 | 1.00 | 0.00 |
| hypothetical protein;Note=hypothetical protein | AFTF01000072.1: 2423-2698 | 1.00 | 1.00 | 1.00 | 1.00 | 1.00 | 1.00 | 1.00 | 1.00 | 1.00 | 0.00 |
| MFS general substrate transporter;Note=MFS general substrate transporter | AFTF01000072.1: 2721-3983 | 1.00 | 1.00 | 1.00 | 1.00 | 1.00 | 1.00 | 1.00 | 1.00 | 1.00 | 0.00 |
| FIG00967211: hypothetical protein;Note=FIG00967211: hypothetical protein | AFTF01000069.1: 12758-13327 | 1.00 | 1.00 | 1.00 | 1.00 | 1.00 | 1.00 | 1.00 | 1.00 | 1.00 | 0.00 |
| ATP binding protein;Note=ATP binding protein | AFTF01000069.1: 13477-14832 | 1.00 | 1.00 | 1.00 | 1.00 | 1.00 | 1.00 | 1.00 | 1.00 | 1.00 | 0.00 |
| Sucrose specific transcriptional regulator CscR2C LacI family;Note=Sucrose specific transcriptional regulator Csc | AFTF01000068.1: 209-1156 | 1.00 | 1.00 | 1.00 | 1.00 | 0.00 | 1.00 | 1.00 | 1.00 | 1.00 | 1.00 |
| Transducer protein car;Note=Transducer protein car | AFTF01000068.1: 1275-6983 | 1.00 | 1.00 | 1.00 | 1.00 | 0.00 | 1.00 | 1.00 | 1.00 | 1.00 | 1.00 |
| hypothetical protein;Note=hypothetical protein | AFTF01000060.1: 1225-1575 | 1.00 | 1.00 | 1.00 | 1.00 | 1.00 | 1.00 | 1.00 | 1.00 | 1.00 | 0.00 |
| hypothetical protein;Note=hypothetical protein | AFTF01000060.1: 1526-1666 | 1.00 | 1.00 | 1.00 | 1.00 | 1.00 | 1.00 | 1.00 | 1.00 | 1.00 | 0.00 |
| FIG00963107: hypothetical protein;Note=FIG00963107: hypothetical protein | AFTF01000409.1: 37033-38406 | 1.00 | 1.00 | 1.00 | 1.00 | 1.00 | 1.00 | 1.00 | 1.00 | 1.00 | 0.00 |
| candidate type III effector HolPtoQ;Note=candidate type III effector HolPtoQ | AFTF01000060.1: 3106-4449 | 1.00 | 1.00 | 1.00 | 1.00 | 1.00 | 1.00 | 1.00 | 1.00 | 1.00 | 0.00 |
| IS1294 orf;Note=IS1294 orf | AFTF01000060.1: 4796-5923 | 0.20 | 0.18 | 0.20 | 0.20 | 1.00 | 1.00 | 1.00 | 0.20 | 0.20 | 0.00 |
| Transcriptional regulator2C LysR family;Note=Transcriptional regulator2C LysR family | AFTF01000059.1: 38238-39137 | 1.00 | 1.00 | 1.00 | 1.00 | 1.00 | 1.00 | 1.00 | 1.00 | 1.00 | 0.00 |
| FIG00966512: hypothetical protein;Note=FIG00966512: hypothetical protein | AFTF01000050.1: 2748-3461 | 0.02 | 0.03 | 0.02 | 0.03 | 1.00 | 1.00 | 1.00 | 0.03 | 0.03 | 0.96 |
| hypothetical protein;Note=hypothetical protein | AFTF01000041.1: 40721-41539 | 1.00 | 1.00 | 1.00 | 1.00 | 1.00 | 1.00 | 1.00 | 1.00 | 1.00 | 0.00 |
| Dipeptide-binding ABC transporter2C periplasmic substrate-binding component (TC 3.A.1.5.2);Note=Dipeptide- | AFTF01000034.1: 561-2138 | 1.00 | 1.00 | 1.00 | 1.00 | 1.00 | 1.00 | 1.00 | 1.00 | 1.00 | 0.00 |
| Dipeptide transport system permease protein DppB (TC 3.A.1.5.2)%3B putative hemin permease;Note=Dipeptid | AFTF01000034.1: 2159-3169 | 1.00 | 1.00 | 1.00 | 1.00 | 1.00 | 1.00 | 1.00 | 1.00 | 1.00 | 0.00 |
| Dipeptide transport system permease protein DppC (TC 3.A.1.5.2);Note=Dipeptide transport system permease | AFTF01000034.1: 3166-4017 | 1.00 | 1.00 | 1.00 | 1.00 | 1.00 | 1.00 | 1.00 | 1.00 | 1.00 | 0.00 |
| Dipeptide transport ATP-binding protein DppD (TC 3.A.1.5.2);Note=Dipeptide transport ATP-binding protein Dp | AFTF01000034.1: 4010-4858 | 1.00 | 1.00 | 1.00 | 1.00 | 1.00 | 1.00 | 1.00 | 1.00 | 1.00 | 0.00 |
| dipeptide ABC transporter2C ATP binding protein;Note=dipeptide ABC transporter2C ATP binding protein | AFTF01000034.1: 4855-5568 | 1.00 | 1.00 | 1.00 | 1.00 | 1.00 | 1.00 | 1.00 | 1.00 | 1.00 | 0.00 |
| Cytochrome B561;Note=Cytochrome B561 | AFTF01000034.1: 5673-6230 | 1.00 | 1.00 | 1.00 | 1.00 | 1.00 | 1.00 | 1.00 | 1.00 | 1.00 | 0.00 |
| transmembrane sensor2C putative;Note=transmembrane sensor2C putative | AFTF01000034.1: 8365-9330 | 1.00 | 1.00 | 1.00 | 1.00 | 1.00 | 1.00 | 1.00 | 1.00 | 1.00 | 0.00 |
| TonB-dependent ferric achromobactin receptor protein;Note=TonB-dependent ferric achromobactin receptor p | AFTF01000034.1: 9400-11802 | 1.00 | 1.00 | 1.00 | 1.00 | 1.00 | 1.00 | 1.00 | 1.00 | 1.00 | 0.00 |
| Shufflon-specific DNA recombinase;Note=Shufflon-specific DNA recombinase | AFTF01000033.1: 33940-34917 | 1.00 | 1.00 | 1.00 | 1.00 | 1.00 | 1.00 | 1.00 | 1.00 | 1.00 | 0.00 |
| hypothetical protein;Note=hypothetical protein | AFTF01000033.1: 34914-35159 | 1.00 | 1.00 | 1.00 | 1.00 | 1.00 | 1.00 | 1.00 | 1.00 | 1.00 | 0.00 |
| hypothetical protein;Note=hypothetical protein | AFTF01000032.1: 13190-13327 | 1.00 | 1.00 | 1.00 | 1.00 | 1.00 | 1.00 | 1.00 | 1.00 | 1.00 | 0.00 |
| hypothetical protein;Note=hypothetical protein | AFTF01000032.1: 37506-37766 | 1.00 | 1.00 | 1.00 | 1.00 | 1.00 | 1.00 | 1.00 | 1.00 | 1.00 | 0.00 |
| Name=Levansucrase;Ontology_term=KEGG_ENZYME:2.4.1.10;Note=Levansucrase Levansucrase;Ontology_tern | AFTF01000031.1: 2809-4104 | 1.00 | 1.00 | 1.00 | 1.00 | 1.00 | 1.00 | 1.00 | 1.00 | 1.00 | 0.00 |
| ParA family protein;Note=ParA family protein | AFTF01000031.1: 17825-18412 | 1.00 | 1.00 | 1.00 | 1.00 | 1.00 | 1.00 | 1.00 | 1.00 | 1.00 | 0.00 |
| FIG00958014: hypothetical protein;Note=FIG00958014: hypothetical protein | AFTF01000028.1: 36172-36435 | 1.00 | 1.00 | 1.00 | 1.00 | 1.00 | 1.00 | 1.00 | 1.00 | 1.00 | 0.00 |

**Supplementary Table 4.** Comparison of gene content among *Psa* isolates based on aligning reads against the reference genome of *Psa* NCPPB3871 (GenBank:AFTF01000000).

| | | | | | | | | | | | |
|---|---|---|---|---|---|---|---|---|---|---|---|
| conserved effector locus protein;Note=conserved effector locus protein | AFTF01000028.1: 53076-53414 | 1.00 | 1.00 | 1.00 | 1.00 | 1.00 | 1.00 | 1.00 | 1.00 | 1.00 | 0.00 |
| type III effector HopN1;Note=type III effector HopN1 | AFTF01000028.1: 53428-54480 | 1.00 | 1.00 | 1.00 | 1.00 | 1.00 | 1.00 | 1.00 | 1.00 | 1.00 | 0.00 |
| FIG00955995: hypothetical protein;Note=FIG00955995: hypothetical protein | AFTF01000026.1: 10075-10281 | 1.00 | 1.00 | 1.00 | 1.00 | 1.00 | 1.00 | 1.00 | 1.00 | 1.00 | 0.00 |
| 3-oxoacyl-[acyl-carrier protein] reductase;Ontology_term=KEGG_ENZYME:1.1.1.100;Note=3-oxoacyl-[acyl-carri | AFTF01000026.1: 10336-11097 | 1.00 | 1.00 | 1.00 | 1.00 | 1.00 | 1.00 | 1.00 | 1.00 | 1.00 | 0.00 |
| bacterial transferase2C hexapeptide repeat protein;Note=bacterial transferase2C hexapeptide repeat protein | AFTF01000026.1: 11109-11735 | 1.00 | 1.00 | 1.00 | 1.00 | 1.00 | 1.00 | 1.00 | 1.00 | 1.00 | 0.00 |
| 3-oxoacyl-[acyl-carrier-protein] synthase2C KASIII;Ontology_term=KEGG_ENZYME:2.3.1.41;Note=3-oxoacyl-[ac | AFTF01000026.1: 11762-12757 | 1.00 | 1.00 | 1.00 | 1.00 | 1.00 | 1.00 | 1.00 | 1.00 | 1.00 | 0.00 |
| major facilitator family transporter;Note=major facilitator family transporter | AFTF01000026.1: 12800-13771 | 1.00 | 1.00 | 1.00 | 1.00 | 1.00 | 1.00 | 1.00 | 1.00 | 1.00 | 0.00 |
| hypothetical protein;Note=hypothetical protein | AFTF01000008.1: 1289-1480 | 0.28 | 0.27 | 0.28 | 0.28 | 1.00 | 1.00 | 1.00 | 0.67 | 0.67 | 0.00 |
| NAD-dependent protein deacetylase of SIR2 family;Note=NAD-dependent protein deacetylase of SIR2 family | AFTF01000397.1: 335-553 | 1.00 | 1.00 | 1.00 | 1.00 | 1.00 | 1.00 | 1.00 | 1.00 | 1.00 | 0.00 |
| Hemolysin activator protein precursor;Note=Hemolysin activator protein precursor | AFTF01000397.1: 1944-3524 | 1.00 | 1.00 | 1.00 | 1.00 | 1.00 | 1.00 | 1.00 | 1.00 | 1.00 | 0.05 |
| Putative large exoprotein involved in heme utilization or adhesion of ShlA/HecA/FhaA family;Note=Putative larg | AFTF01000397.1: 3549-12383 | 1.00 | 1.00 | 1.00 | 1.00 | 1.00 | 1.00 | 1.00 | 1.00 | 1.00 | 0.02 |
| Error-prone2C lesion bypass DNA polymerase V (UmuC);Note=Error-prone2C lesion bypass DNA polymerase V ( | AFTF01000396.1: 3594-3842 | 0.01 | 0.00 | 0.02 | 0.02 | 1.00 | 1.00 | 1.00 | 0.31 | 0.31 | 0.00 |
| Uncharacterized protein ImpA;Note=Uncharacterized protein ImpA | AFTF01000359.1: 434-1765 | 0.04 | 0.04 | 0.05 | 0.05 | 1.00 | 1.00 | 1.00 | 0.10 | 0.10 | 1.00 |
| hypothetical protein;Note=hypothetical protein | AFTF01000358.1: 20-145 | 0.55 | 0.60 | 0.56 | 0.72 | 1.00 | 1.00 | 1.00 | 0.72 | 0.61 | 0.00 |
| transcriptional regulator2C LysR family;Note=transcriptional regulator2C LysR family | AFTF01000356.1: 252-1076 | 1.00 | 1.00 | 1.00 | 1.00 | 0.00 | 1.00 | 1.00 | 1.00 | 1.00 | 1.00 |
| FIG00954449: hypothetical protein;Note=FIG00954449: hypothetical protein | AFTF01000356.1: 1216-3966 | 1.00 | 1.00 | 1.00 | 1.00 | 0.00 | 1.00 | 1.00 | 1.00 | 1.00 | 1.00 |
| hypothetical protein;Note=hypothetical protein | AFTF01000353.1: 446-619 | 0.59 | 0.58 | 0.59 | 0.60 | 1.00 | 1.00 | 1.00 | 0.59 | 0.59 | 0.00 |
| hypothetical protein;Note=hypothetical protein | AFTF01000352.1: 84-566 | 0.06 | 0.05 | 0.06 | 0.06 | 1.00 | 1.00 | 1.00 | 0.05 | 0.05 | 0.00 |
| hypothetical protein;Note=hypothetical protein | AFTF01000348.1: 24-437 | 0.53 | 0.61 | 0.70 | 0.72 | 0.20 | 1.00 | 0.20 | 0.82 | 0.81 | 0.00 |
| TRAP-type C4-dicarboxylate transport system2C periplasmic component;Note=TRAP-type C4-dicarboxylate trar | AFTF01000344.1: 1185-1697 | 0.04 | 0.05 | 0.05 | 0.05 | 1.00 | 1.00 | 1.00 | 0.14 | 0.14 | 1.00 |
| hypothetical protein;Note=hypothetical protein | AFTF01000343.1: 1524-2936 | 0.01 | 0.01 | 0.02 | 0.02 | 1.00 | 1.00 | 1.00 | 0.12 | 0.12 | 0.02 |
| hypothetical protein;Note=hypothetical protein | AFTF01000340.1: 77-262 | 0.02 | 0.13 | 0.10 | 0.12 | 0.24 | 1.00 | 1.00 | 0.18 | 0.16 | 0.00 |
| Two-component response regulator;Note=Two-component response regulator | AFTF01000336.1: 150-1655 | 1.00 | 1.00 | 1.00 | 1.00 | 1.00 | 1.00 | 1.00 | 1.00 | 1.00 | 0.00 |
| ATP-dependent helicase HrpB;Note=ATP-dependent helicase HrpB | AFTF01000330.1: 1092-2456 | 0.62 | 0.62 | 0.62 | 0.62 | 1.00 | 1.00 | 1.00 | 0.68 | 0.68 | 0.03 |
| FIG00953710: hypothetical protein;Note=FIG00953710: hypothetical protein | AFTF01000329.1: 1083-1343 | 0.07 | 0.04 | 0.07 | 0.10 | 0.10 | 1.00 | 1.00 | 0.11 | 0.11 | 1.00 |
| hypothetical protein;Note=hypothetical protein | AFTF01000322.1: 2533-3027 | 1.00 | 1.00 | 1.00 | 1.00 | 1.00 | 1.00 | 1.00 | 1.00 | 1.00 | 0.03 |
| FIG00454553: hypothetical protein;Note=FIG00454553: hypothetical protein | AFTF01000316.1: 113-3364 | 0.01 | 0.01 | 0.01 | 0.01 | 1.00 | 1.00 | 1.00 | 0.05 | 0.05 | 0.02 |
| hypothetical protein;Note=hypothetical protein | AFTF01000313.1: 1289-1501 | 1.00 | 1.00 | 1.00 | 1.00 | 0.00 | 0.00 | 1.00 | 1.00 | 1.00 | 0.00 |
| FIG00955572: hypothetical protein;Note=FIG00955572: hypothetical protein | AFTF01000307.1: 760-2493 | 0.01 | 0.01 | 0.05 | 0.01 | 1.00 | 1.00 | 1.00 | 0.02 | 0.02 | 0.01 |
| Name=Integrase Integrase;Note=Integrase | AFTF01000305.1: 109-1419 | 0.00 | 0.00 | 0.01 | 0.01 | 0.87 | 1.00 | 0.00 | 0.01 | 0.01 | 0.00 |
| SanC2C putative;Note=SanC2C putative | AFTF01000299.1: 665-1036 | 1.00 | 1.00 | 1.00 | 1.00 | 1.00 | 1.00 | 1.00 | 1.00 | 1.00 | 0.00 |
| FIG00959382: hypothetical protein;Note=FIG00959382: hypothetical protein | AFTF01000299.1: 3653-4573 | 1.00 | 1.00 | 1.00 | 1.00 | 1.00 | 1.00 | 1.00 | 1.00 | 1.00 | 0.00 |
| plasmid stability protein StbB;Note=plasmid stability protein StbB | AFTF01000299.1: 6937-7083 | 1.00 | 1.00 | 1.00 | 1.00 | 1.00 | 1.00 | 1.00 | 1.00 | 1.00 | 0.00 |
| plasmid stability protein StbC;Note=plasmid stability protein StbC | AFTF01000299.1: 7080-7202 | 1.00 | 1.00 | 1.00 | 1.00 | 1.00 | 1.00 | 1.00 | 1.00 | 1.00 | 0.00 |
