# Supplementary material for: Pseudomonas syringae pv. actinidiae (PSA) Isolates from Recent Bacterial Canker of Kiwifruit Outbreaks Belong to the Same Genetic Lineage
Source: PLoS One. 2012 May 9;7(5):e36518. doi: 10.1371/journal.pone.0036518 (PMC3348921; doi:10.1371/journal.pone.0036518)
Supplement: Table S5 — Gene contents of isolates CFBP7286 (Europe) and CH2010-6 (China) in the region of the PPHGI-1- like island based on aligning reads against the reference genome of PSA MAFF302091. (PDF) [file pone.0036518.s005.pdf]

Supplementary Table 5. Gene contents of isolates CFBP7286 (Europe) and CH2010-6 (China) in the region of the PPHGI-1-like island based on aligning reads against the reference genome of *Psa* MAFF302091.

Gene	Location in MAFF 302091	CFBP7286	CH2010-6
As predicted by RAST (http://http://rast.nmpdr.org/)	(GenBank accession: start-end)	Italy	China
RlgA	AEAL01000941.1: 166-546	1.00	1.00
transcriptional regulator2C LysR family	AEAL01000643.1: 2894-3718	1.00	1.00
Transposase	AEAL01000934.1: 47-238	0.28	0.67
TonB-dependent ferric achromobactin receptor protein	AEAL01000447.1: 6907-9309	1.00	1.00
transmembrane sensor2C putative	AEAL01000447.1: 9379-10344	1.00	1.00
Cytochrome B561	AEAL01000447.1: 12479-13036	1.00	1.00
Putative glutathione transporter2C ATP-binding component	AEAL01000447.1: 13141-13854	1.00	1.00
Dipeptide transport ATP-binding protein DppD (TC 3.A.1.5.2)	AEAL01000447.1: 13851-14699	1.00	1.00
Dipeptide transport system permease protein DppC (TC 3.A.1.5.2)	AEAL01000447.1: 14692-15543	1.00	1.00
Dipeptide transport system permease protein DppB (TC 3.A.1.5.2)%3B putati	AEAL01000447.1: 15540-16550	1.00	1.00
Dipeptide-binding ABC transporter2C periplasmic substrate-binding compon	AEAL01000447.1: 16571-18148	1.00	1.00
avirulence protein	AEAL01000419.1: 368-1387	1.00	1.00
hypothetical protein	AEAL01000419.1: 2072-2215	1.00	1.00
hypothetical protein	AEAL01000419.1: 2595-2753	1.00	1.00
transposase	AEAL01000419.1: 2743-3153	1.00	1.00
hypothetical protein	AEAL01000419.1: 3112-3285	1.00	1.00
hypothetical protein	AEAL01000418.1: 816-1028	1.00	1.00
Methyl-accepting chemotaxis protein	AEAL01000416.1: 643-1017	1.00	1.00
putative DNA helicase	AEAL01000416.1: 1075-2481	1.00	1.00
Aconitase B	AEAL01000416.1: 2491-2820	1.00	1.00
hypothetical protein	AEAL01000415.1: 1052-1213	0.96	0.97
Sucrose specific transcriptional regulator CscR2C LacI family	AEAL01000414.1: 80-1075	1.00	1.00
Transducer protein car	AEAL01000414.1: 1194-6902	1.00	1.00
transposase	AEAL01000413.1: 44-250	1.00	1.00
macrolide-efflux protein	AEAL01000413.1: 558-1820	1.00	1.00
hypothetical protein	AEAL01000413.1: 1843-2118	1.00	1.00
hypothetical protein	AEAL01000407.1: 5967-6473	1.00	1.00
FIG00635456: hypothetical protein	AEAL01000407.1: 6466-7362	1.00	1.00
ParA family protein	AEAL01000388.1: 662-943	1.00	1.00
Levansucrase (EC 2.4.1.10);Ontology_term=KEGG_ENZYME:2.4.1.10	AEAL01000384.1: 2764-4059	1.00	1.00
**Protein with ParB-like nuclease domain in PFGI-1-like cluster**	**AEAL01000369.1: 6-1274**	**0.02**	**0.84**
**FIG004780: hypothetical protein in PFGI-1-like cluster**	**AEAL01000369.1: 1311-2048**	**0.07**	**0.96**
**DNA/RNA helicase in PFGI-1-like cluster**	**AEAL01000369.1: 8387-10381**	**0.04**	**0.84**
Conjugative transfer protein PilL in PFGI-1-like cluster	AEAL01000369.1: 11925-13070	0.04	0.28
Conjugative transfer protein PilN in PFGI-1-like cluster	AEAL01000369.1: 13067-14803	0.04	0.13
Plasmid stability protein stbB	AEAL01000333.1: 1398-1544	1.00	1.00
Plasmid stability protein stbC	AEAL01000333.1: 1541-1663	1.00	1.00
SanC2C putative	AEAL01000332.1: 469-840	1.00	1.00
FIG00959378: hypothetical protein	AEAL01000332.1: 3457-4377	1.00	1.00
putative integrase protein	AEAL01000941.1: 530-2188	1.00	1.00
Unknown2C probable hemolysin/adhesin	AEAL01000603.1: 32-6979	0.99	0.99
VgrG protein	AEAL01000317.1: 40708-42735	1.00	1.00
avirulence protein	AEAL01000312.1: 16469-16915	1.00	1.00
site-specific recombinase2C phage integrase family	AEAL01000311.1: 1070-2080	1.00	1.00
Serine protease	AEAL01000311.1: 2058-3113	0.08	0.08
FIG00958716: hypothetical protein	AEAL01000309.1: 111-719	0.52	0.62
Serine protease	AEAL01000309.1: 2600-4696	0.06	0.08
hypothetical protein	AEAL01000307.1: 49-171	1.00	1.00
FIG00961386: hypothetical protein	AEAL01000306.1: 1534-1884	1.00	1.00
FIG00961386: hypothetical protein	AEAL01000306.1: 1997-2341	1.00	1.00
hypothetical protein	AEAL01000814.1: 103-225	0.89	0.96
Hemolysin	AEAL01000595.1: 3352-12186	1.00	1.00
FIG00965775: hypothetical protein	AEAL01000794.1: 186-332	1.00	1.00
hypothetical protein	AEAL01000592.1: 1167-1292	1.00	1.00
hypothetical protein	AEAL01000281.1: 13057-13194	1.00	1.00
chromosome segregation ATPase	AEAL01000591.1: 49-1113	0.66	0.66
hypothetical protein	AEAL01000281.1: 37372-37632	1.00	1.00
membrane protein2C putative	AEAL01000279.1: 1763-2290	1.00	1.00
FIG00964546: hypothetical protein	AEAL01000279.1: 2416-3252	1.00	1.00
ABC-type polar amino acid transport system2C ATPase component	AEAL01000276.1: 6929-7726	1.00	1.00
ABC-type amino acid transport/signal transduction systems2C periplasmic co	AEAL01000276.1: 9108-9461	1.00	1.00

Supplementary Table 5. Gene contents of isolates CFBP7286 (Europe) and CH2010-6 (China) in the region of the PPHGI-1-like island based on aligning reads against the reference genome of *Psa* MAFF302091.

ABC-type amino acid transport/signal transduction systems2C periplasmic co	AEAL01000276.1: 9680-9979	● 1.00	● 1.00
Cellulose synthase operon protein C	AEAL01000269.1: 272-4087	◑ 0.57	◑ 0.57
putative inner membrane protein	AEAL01000263.1: 72-563	● 0.94	● 0.94
YoeB toxin protein	AEAL01000258.1: 17171-17473	● 1.00	● 1.00
Colicin immunity protein	AEAL01000250.1: 39-302	● 1.00	● 1.00
candidate type III effector HolPtoQ	AEAL01000242.1: 132-1475	● 1.00	● 1.00
hypothetical protein	AEAL01000242.1: 2915-3055	● 1.00	● 1.00
hypothetical protein	AEAL01000242.1: 3006-3356	● 1.00	● 1.00
hypothetical protein	AEAL01000231.1: 408-548	● 1.00	● 1.00
protein of unknown function DUF1526	AEAL01000231.1: 532-1002	● 1.00	● 1.00
FIG045374: Type II restriction enzyme2C methylase subunit YeeA	AEAL01000200.1: 1307-3379	● 1.00	● 1.00
Protein involved in meta-pathway of phenol degradation	AEAL01000200.1: 3364-4257	○ 0.05	○ 0.05
hypothetical protein	AEAL01000188.1: 40780-40899	● 1.00	● 1.00
ATP binding protein	AEAL01000180.1: 50-1561	● 1.00	● 1.00
FIG00963102: hypothetical protein	AEAL01000144.1: 165-1538	● 1.00	● 1.00
hypothetical protein	AEAL01000140.1: 1432-1980	● 1.00	● 1.00
hypothetical protein	AEAL01000138.1: 1619-1762	● 1.00	● 1.00
hypothetical protein	AEAL01000138.1: 1799-2344	● 1.00	● 1.00
NreA-like protein	AEAL01000138.1: 2573-2866	● 1.00	● 1.00
Nickel and cobalt efflux transporter rcnA	AEAL01000138.1: 2874-3746	● 1.00	● 1.00
hypothetical protein	AEAL01000119.1: 128-292	● 1.00	● 1.00
Putative heat shock protein YegD	AEAL01000119.1: 842-2407	● 1.00	● 1.00
type III effector AvrB4-2	AEAL01000939.1: 1249-2211	● 1.00	● 1.00
Transcriptional regulator2C LysR family	AEAL01000068.1: 37950-38849	● 1.00	● 1.00
Unknown pentose kinase TM0952	AEAL01000063.1: 11806-13299	● 0.92	● 0.95
Transketolase2C C-terminal section (EC 2.2.1.1);Ontology_term=KEGG_ENZY	AEAL01000063.1: 13312-14244	● 1.00	● 1.00
Transketolase2C N-terminal section (EC 2.2.1.1);Ontology_term=KEGG_ENZY	AEAL01000063.1: 14234-15220	● 1.00	● 1.00
Xylose ABC transporter2C permease protein XylH	AEAL01000063.1: 15434-16015	● 1.00	● 1.00
Xylose ABC transporter2C permease protein XylH	AEAL01000063.1: 16016-16438	● 1.00	● 1.00
Xylose ABC transporter2C periplasmic xylose-binding protein XylF	AEAL01000063.1: 16505-17437	● 1.00	● 1.00
D-xylose transport ATP-binding protein XylG	AEAL01000063.1: 17453-18985	● 1.00	● 1.00
Short chain dehydrogenase	AEAL01000063.1: 19017-19781	● 1.00	● 1.00
SHORT CHAIN DEHYDROGENASE	AEAL01000063.1: 19812-20612	● 1.00	● 1.00
Putative transcriptional regulator of sorbose uptake and utilization genes	AEAL01000063.1: 20694-21647	● 0.99	● 1.00
titin	AEAL01000061.1: 106-1275	● 1.00	● 1.00
RecA/RadA recombinase	AEAL01000061.1: 1250-1873	● 1.00	● 1.00
SOS-response repressor and protease LexA (EC 3.4.21.88);Ontology_term=KE	AEAL01000061.1: 1870-2487	● 1.00	● 1.00
titin	AEAL01000060.1: 805-2034	● 1.00	● 1.00
DNA polymerase III alpha subunit (EC 2.7.7.7);Ontology_term=KEGG_ENZYM	AEAL01000060.1: 2092-2856	● 1.00	● 1.00
DNA polymerase-like protein PA0670	AEAL01000060.1: 2853-3032	● 1.00	● 1.00
hypothetical protein	AEAL01000057.1: 264-509	● 1.00	● 1.00
Shufflon-specific DNA recombinase	AEAL01000057.1: 506-1483	● 1.00	● 1.00
hypothetical protein	AEAL01000038.1: 8424-8687	● 1.00	● 1.00
type III effector HopPtoN	AEAL01000004.1: 713-1621	● 1.00	● 1.00
conserved effector locus protein	AEAL01000004.1: 1779-2258	● 1.00	● 1.00
FIG00958014: hypothetical protein	AEAL01000004.1: 18757-19020	● 1.00	● 1.00
hypothetical protein	AEAL01000001.1: 695-868	◑ 0.59	◑ 0.59
transcriptional regulator2C AraC family protein	AEAL01000538.1: 85-930	● 1.00	● 1.00
ABC-type amino acid transport/signal transduction systems2C periplasmic co	AEAL01000538.1: 2008-2259	● 1.00	● 1.00
ABC-type amino acid transport/signal transduction systems2C periplasmic co	AEAL01000538.1: 2314-2880	● 1.00	● 1.00
Glutamate transport membrane-spanning protein	AEAL01000538.1: 3635-4294	● 1.00	● 1.00
Glutamate transport membrane-spanning protein	AEAL01000538.1: 4275-4940	● 1.00	● 1.00
FIG00962023: hypothetical protein	AEAL01000538.1: 5250-5411	● 1.00	● 1.00
transcriptional regulator2C AraC family	AEAL01000537.1: 10018-11001	● 1.00	● 1.00
hypothetical protein	AEAL01000537.1: 11211-11390	● 1.00	● 1.00
Transcriptional regulator2C TetR family	AEAL01000537.1: 12505-13086	● 1.00	● 1.00
NADH:flavin oxidoreductases2C Old Yellow Enzyme family	AEAL01000537.1: 13142-13543	◕ 0.65	◕ 0.66
NADH:flavin oxidoreductases2C Old Yellow Enzyme family	AEAL01000537.1: 13584-14252	● 1.00	● 1.00
Permeases of the major facilitator superfamily	AEAL01000537.1: 14310-14696	● 1.00	● 1.00
oxidoreductase ylbE	AEAL01000537.1: 14757-15452	● 1.00	● 1.00
NADH:flavin oxidoreductases2C Old Yellow Enzyme family	AEAL01000537.1: 15430-15612	● 1.00	● 1.00
FIG00961033: hypothetical protein	AEAL01000536.1: 543-1253	◕ 0.77	● 0.83
FIG00959133: hypothetical protein	AEAL01000536.1: 1250-1786	◕ 0.80	● 0.82

Supplementary Table 5. Gene contents of isolates CFBP7286 (Europe) and CH2010-6 (China) in the region of the PPHGI-1-like island based on aligning reads against the reference genome of *Psa* MAFF302091.

FIG00958203: hypothetical protein	AEAL01000536.1: 1783-2709	● 0.94	● 0.99
FIG00958486: hypothetical protein	AEAL01000536.1: 2706-3149	● 1.00	● 1.00
Phage tail fiber protein	AEAL01000536.1: 3306-5750	● 1.00	● 1.00
FIG00953961: hypothetical protein	AEAL01000536.1: 5759-6283	● 0.98	● 0.98
Putative bacteriophage protein	AEAL01000536.1: 6280-7449	● 1.00	● 1.00
FIG00953163: hypothetical protein	AEAL01000536.1: 7446-7763	● 0.96	● 1.00
Phage-related tail protein	AEAL01000536.1: 7763-10063	● 0.81	● 0.82
FIG00958553: hypothetical protein	AEAL01000536.1: 10069-10212	● 1.00	● 1.00
FIG00958914: hypothetical protein	AEAL01000536.1: 10233-10523	● 1.00	● 1.00
Endopeptidase (EC 3.4.-.-);Ontology_term=KEGG_ENZYME:3.4.-.-	AEAL01000536.1: 10557-11036	● 0.91	● 0.92
putative phage lysozyme	AEAL01000536.1: 11033-11560	● 1.00	● 1.00
FIG00964753: hypothetical protein	AEAL01000536.1: 11557-11763	● 1.00	● 1.00
Hypothetical Zinc-finger containing protein	AEAL01000536.1: 11760-11972	● 1.00	● 1.00
FIG00961890: hypothetical protein	AEAL01000536.1: 11972-12424	● 0.99	● 0.99
Conserved hypothetical phage protein	AEAL01000536.1: 12428-13540	● 0.95	● 0.97
FIG00956268: hypothetical protein	AEAL01000536.1: 13555-14223	◕ 0.62	◑ 0.53
FIG00955158: hypothetical protein	AEAL01000536.1: 14213-14656	● 0.87	● 0.87
Probable head completion/stabilization protein	AEAL01000536.1: 14653-15114	● 1.00	● 1.00
hypothetical protein	AEAL01000536.1: 15150-15446	● 1.00	● 1.00
hypothetical protein	AEAL01000536.1: 15940-16239	● 1.00	● 1.00
FIG00967513: hypothetical protein	AEAL01000536.1: 16379-17101	● 1.00	● 1.00
Phage-related capsid packaging protein	AEAL01000536.1: 21533-22048	● 0.97	● 0.98
hypothetical protein	AEAL01000536.1: 22244-22384	● 0.87	● 0.89
hypothetical protein	AEAL01000536.1: 23636-23890	● 1.00	● 1.00
hypothetical protein	AEAL01000536.1: 24329-24577	◑ 0.59	◑ 0.60
prophage PSPPH062C site-specific recombinase2C phage integrase family	AEAL01000536.1: 24540-25025	● 1.00	● 1.00
hypothetical protein	AEAL01000536.1: 25277-25567	● 1.00	● 1.00
Glucans biosynthesis protein G precursor	AEAL01000534.1: 1121-2707	● 1.00	● 1.00
Two-component response regulator	AEAL01000533.1: 42-1508	● 1.00	● 1.00
ATP-dependent helicase HrpB	AEAL01000521.1: 2110-4209	○ 0.03	○ 0.03
hypothetical protein	AEAL01000937.1: 326-520	● 1.00	● 1.00
MULTIDRUG RESISTANCE PROTEIN 1	AEAL01000502.1: 387-1358	● 1.00	● 1.00
3-oxoacyl-[acyl-carrier-protein] synthase2C KASIII (EC 2.3.1.41);Ontology_ter	AEAL01000502.1: 1401-2396	● 1.00	● 1.00
bacterial transferase2C hexapeptide repeat protein	AEAL01000502.1: 2423-3049	● 1.00	● 1.00
Dehydrogenases with different specificities (related to short-chain alcohol de	AEAL01000502.1: 3061-3822	● 1.00	● 1.00
FIG00955995: hypothetical protein	AEAL01000502.1: 3877-4083	● 1.00	● 1.00
ABC-type amino acid transport2C signal transduction systems2C periplasmic	AEAL01000496.1: 10961-11716	○ 0.04	○ 0.04
FIG00962378: hypothetical protein	AEAL01000495.1: 435-1385	● 1.00	● 1.00
hypothetical protein	AEAL01000650.1: 781-915	● 1.00	● 1.00
